# Inverse regulation of light harvesting and photoprotection is mediated by a 3′-end-derived sRNA in cyanobacteria

**DOI:** 10.1093/plcell/koaa030

**Published:** 2020-12-14

**Authors:** Jiao Zhan, Claudia Steglich, Ingeborg Scholz, Wolfgang R Hess, Diana Kirilovsky

**Affiliations:** 1 Université Paris-Saclay, Commissariat à l’Énergie Atomiques et aux Énergies Alternatives, Centre National de la Recherche Scientifique (CEA, CNRS), Institute for Integrative Biology of the Cell (I2BC), 91198 Gif sur Yvette, France; 2 Key Laboratory of Algal Biology, Institute of Hydrobiology, Chinese Academy of Sciences, Wuhan 430072, China; 3 Faculty of Biology, Institute of Biology III, University of Freiburg, D-79104 Freiburg im Breisgau, Germany

## Abstract

Phycobilisomes (PBSs), the principal cyanobacterial antenna, are among the most efficient macromolecular structures in nature, and are used for both light harvesting and directed energy transfer to the photosynthetic reaction center. However, under unfavorable conditions, excess excitation energy needs to be rapidly dissipated to avoid photodamage. The orange carotenoid protein (OCP) senses light intensity and induces thermal energy dissipation under stress conditions. Hence, its expression must be tightly controlled; however, the molecular mechanism of this regulation remains to be elucidated. Here, we describe the discovery of a posttranscriptional regulatory mechanism in *Synechocystis* sp. PCC 6803 in which the expression of the operon encoding the allophycocyanin subunits of the PBS is directly and in an inverse fashion linked to the expression of OCP. This regulation is mediated by ApcZ, a small regulatory RNA that is derived from the 3′-end of the tetracistronic *apcABC–apcZ* operon. ApcZ inhibits *ocp* translation under stress-free conditions. Under most stress conditions, *apc* operon transcription decreases and *ocp* translation increases. Thus, a key operon involved in the collection of light energy is functionally connected to the expression of a protein involved in energy dissipation. Our findings support the view that regulatory RNA networks in bacteria evolve through the functionalization of mRNA 3′-UTRs.

## Teaser

A posttranscriptional regulatory mechanism links the expression of the *Synechocystis* Orange Carotenoid Protein directly and in an inverse fashion to phycobilisome synthesis via a 3′-end-derived sRNA.

## Introduction

In all forms of life, extensive regulatory systems have evolved that enable organisms to acclimate to adverse environmental conditions. Photosynthetic organisms, which depend on light for survival and environmental cues, must constantly sense changes in light quality and quantity and have therefore developed diverse mechanisms enabling them to thrive in fluctuating environments. Regulatory, noncoding RNA molecules are often involved in acclimation to varying environmental conditions. For example, in plants, microRNAs (miRNAs) and small interfering RNAs (siRNAs), which represent two major classes of small RNA (sRNA) regulators, exert important controls both in developmental regulation and in stress responses (reviewed in Khraiwesh et al., 2012; Meyers and Axtell, 2019; Song et al., 2019). Noncoding RNAs were also discovered in plant chloroplasts (Lung et al., 2006; Hotto et al., 2011; Zhelyazkova et al., 2012; Ruwe et al., 2016), but their regulatory potential has not been fully elucidated yet. In view of these findings, it appears striking that reports on regulatory RNAs specifically affecting photosynthesis-related processes are scarce. Therefore, we set out to characterize such RNA regulators in photosynthetic cyanobacteria, the direct evolutionary progenitors of chloroplasts.

The rationale for this has been that, in bacteria, dozens of sRNAs have been recognized unambiguously as essential posttranscriptional regulators (Storz et al., 2011) and that hundreds of additional sRNAs still await functional characterization (for reviews, see Kopf and Hess [2015] and Adams and Storz [2020]). Indeed, in cyanobacteria, several regulatory sRNAs which impact the photosynthetic apparatus have been described. The sRNA PsrR1 was demonstrated to regulate several photosynthetic genes during exposure to high light intensities (confirmed for at least *psaJ*, *psaL*, *psbB*, and *cpcA*; Georg et al., 2014) and the sRNA IsaR1 to control the expression of more than 15 genes during iron starvation. Target genes that were unambiguously assigned to IsaR1 encode the major ferredoxin (Fed1), cytochrome *c_6_* (PetJ), Fe/S biogenesis proteins SufBCDS, the superoxide dismutase subunit SodB, the cytochrome *b_6_f* complex proteins PetABDC1, aconitate hydratase (AcnB), and the tetrapyrrole biosynthesis enzymes HemA and ChlN (Georg et al., 2017).

Photosynthetic organisms can acclimate to adverse environmental conditions also by adapting their photosynthetic apparatus. Flexible mechanisms for increasing heat dissipation of excess absorbed excitation energy help to avoid photodamage under high light conditions but secure efficient light harvesting under moderate and low light intensities. In cyanobacteria, a water-soluble photoactive carotenoid protein, the orange carotenoid protein (OCP), senses light intensity and induces thermal dissipation of excess excitation energy by interacting with the phycobilisome (PBS), the cyanobacterial antenna (see reviews Kirilovsky and Kerfeld, 2016; Sluchanko et al., 2017; Muzzopappa and Kirilovsky, 2019).

**Figure 1:**
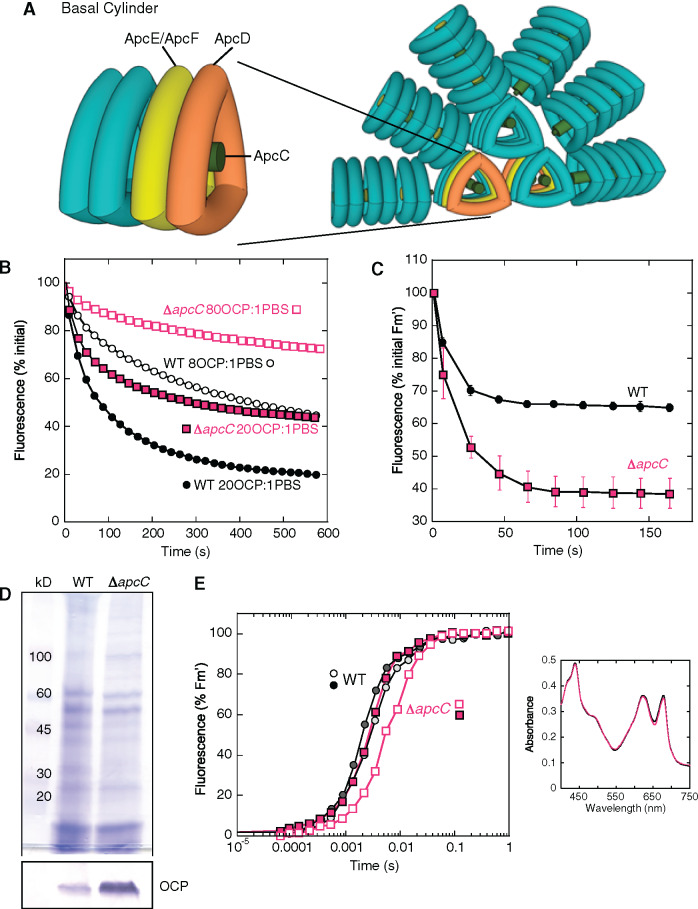
Effect of the lack of ApcC in *Synechocystis* cells. (A) The *Synechocystis* PBS. Six PC rods radiate from the core formed by three APC cylinders. A basal cylinder is shown where the position of the trimers containing ApcD or ApcE/ApcF is indicated. The position of ApcC is also given. (B) Purified PBSs (0.012 µM) from WT (black) and Δ*apcC* mutant (fuchsia) were incubated with two different concentrations of pre-photoactivated OCP giving a final ratio of 20 (closed symbols) or 8 (open symbols) OCP per PBS. The decrease of fluorescence induced in 0.5 M phosphate buffer at 23°C under strong blue light (900 μmol m^−2^ s^−1^) is shown (three biologically independent experiments). The error bars are not shown for clarity. (C) Decrease of maximal fluorescence induced by strong blue light in cells. WT (black) and Δ*apcC* (fuchsia) *Synechocystis* cells were adapted to low blue light (80 µmol photons s^−1^ m^−2^) and then illuminated with strong blue light (1,200 µmol photons s^−1^ m^−2^) causing a decrease in fluorescence (Fm′). The ratio PC to chlorophyll was similar in WT and Δ*apcC* cells. Three biologically independent experiments were performed. The error bars represent sd. The complete PAM traces are shown in Supplemental Figure S1. (D) Coomassie Brilliant Blue and immunoblot detection of OCP in membrane-PBS fractions purified from WT and Δ*apcC* mutant. For each lane, 2 µg of chlorophyll was loaded. The detection of OCP in other PBS mutants is shown in Supplemental Figure S2. (E) Induction of fluorescence in dark-adapted (closed symbols) and in high-light exposed (open symbols) WT (black squares) and Δ*apcC* mutant (fuchsia circles) cells in the presence of DCMU (10 µM) and DBMIB (10 µM). A representative experiment is shown. The measurements were repeated with three independent biological replicates. Inset: Absorbance spectra of WT (black) and mutant (fuchsia) cells used in this experiment. The ratio PC to chlorophyll was similar in WT and Δ*apcC* cells.

The PBS is a huge extramembrane complex formed by a core attached to the stromal side of thylakoids, from which rods radiate (see reviews Adir, 2008; Watanabe and Ikeuchi, 2013; Adir et al., 2019). The core and rods contain light-absorbing phycobiliproteins and linker proteins, which principally have a structural role. In *Synechocystis* sp. PCC 6803 (hereafter *Synechocystis*), the cyanobacterium used in this study, each rod is composed of three phycocyanin (PC) hexamers, and the core contains three allophycocyanin (APC) cylinders from which six rods radiate (Arteni et al., 2009; Figure 1, A). Both PC and APC covalently bind the blue bilin, phycocyanobilin. The upper cylinder contains four APC trimers formed by αAPC–βAPC heterodimers, which have a fluorescence maximum at 660 nm. In the basal cylinders, in two of the trimers, one α and/or one β subunit, are replaced by ApcD, ApcF, or the bilin-linker domain of ApcE. The trimers containing ApcD and ApcE/ApcF emit at 680 nm and transfer the excitation energy to the photosystems. ApcE is also essential for the integrity of the PBS core and the interaction of the PBS with the membrane (Redlinger and Gantt, 1982). All the external trimers of the cylinders contain the linker-core protein, Lc, or ApcC, in the central hole (Figure 1, A).

OCP is a modular protein formed by two domains, the N-terminal domain (NTD), which is the effector domain, and the C-terminal domain (CTD), which is the regulator domain (see reviews Kirilovsky and Kerfeld, 2016; Kerfeld et al., 2017; Sluchanko et al., 2017; Muzzopappa and Kirilovsky, 2019). In the dark inactive orange form (OCP^O^), the two domains have strong interactions, and the protein has a closed conformation (Kerfeld et al., 2003). The two domains share a ketocarotenoid molecule, the hydroxyechinenone, hECN, which is stabilized by hydrogen bonds between the carotenoid carbonyl and a Tyr and a Trp residue in the CTD (Kerfeld et al., 2003). Upon absorption of strong blue light, these hydrogen bonds are broken, and the carotenoid moves 12 Å into the NTD (Leverenz et al., 2015). In the photoactivated OCP (OCP^R^), which is red and the active form (Wilson et al., 2008), the domains separate (Gupta et al., 2015). The carotenoid in the NTD is more planar and has a longer conjugation length than in OCP^O^. The NTD is then able to interact with the core of the PBS and induce heat dissipation of excess energy (Wilson et al., 2008; Gwizdala et al., 2011; Wilson et al., 2012; Leverenz et al., 2014; Harris et al., 2016). The heat dissipation is accompanied by a decrease (quenching) in PBS fluorescence (Wilson et al., 2006).

Three types of OCP were described: OCP1, OCP2, and OCPX (Bao et al., 2017; Muzzopappa et al., 2019). Most cyanobacteria contain only OCP1 or OCPX, but some strains also contain OCP2 (Bao et al., 2017; Muzzopappa et al., 2019). OCP2 is specifically expressed under stress conditions (Bao et al., 2017), whereas OCP1 and OCPX are always present in the cells, although in varying concentrations according to the environmental conditions (Wilson et al., 2006, 2007). *Synechocystis* contains only OCP1 that is constitutively expressed at a basal level (Singh et al., 2004; Wilson et al., 2006, 2007; Yingping et al., 2014) but is increased further under high light (Hihara et al., 2001), iron starvation (Singh et al., 2004; Wilson et al., 2007; Yingping et al., 2014), oxidative stress (Singh et al., 2004; Yingping et al., 2014), UV light (Huang et al., 2002), osmotic stress (Paithoonrangsarid et al., 2004), or after exposure to 2,5-dibromo-3-methyl-6-isopropyl *p*-benzoquinone (DBMIB; Hihara et al., 2003), indicating sensitivity to changes in the redox status of the photosynthetic electron transport chain. Its expression is also enhanced under severe intracellular C_i_ limitation, whereas the accumulation of PBS-associated transcripts is downregulated (Orf et al., 2015). An integration of OCP expression with the regulation of photosynthesis- and PBS-associated genes appears consequential. However, almost nothing is known about how such a hypothetical regulatory mechanism could operate or how OCP transcription and transcript accumulation are regulated.

Here, we describe the discovery of a posttranscriptional regulatory mechanism in which the expression of the *apcABC* operon is directly and in an inverse fashion linked to the expression of OCP. The critical molecule in this mechanism is the 3′-end-derived sRNA ApcZ that connects the expression of OCP and PBS genes in a surprisingly simple but elegant way.

## Results

### The unexpected phenotype of the Δ*apcC* mutant

A few years ago, we constructed several *Synechocystis* mutants lacking one of the PBS core proteins, and we characterized the OCP-related photoprotective mechanism in these strains (Jallet et al., 2012). We showed that the absence of ApcD or ApcF or the modification of the bilin-binding site in ApcE had no influence on the amplitude and kinetics of blue-light-induced quenching in vivo and in vitro (Jallet et al., 2012). In contrast, more recently, we observed that the lack of the core linker ApcC led to a smaller amplitude of PBS fluorescence quenching when purified PBSs were illuminated in the presence of OCP (Harris et al., 2016). This phenomenon was explained as a result of weaker OCP binding to the PBS core in the absence of ApcC (Harris et al., 2016). These in vitro results were confirmed in the present work as shown in Figure 1, B. Purified wild-type (WT) PBSs and PBSs lacking ApcC were illuminated in the presence of two OCP concentrations giving final ratios of 20 and 8 OCP per PBS. Consistent with the previous observations, the amplitude of fluorescence quenching induced by strong blue–green light was larger in WT PBS than in the PBS lacking ApcC. Then, we wanted to confirm these results in vivo.

The induction, kinetics, and amplitude of the OCP-related photoprotective mechanism in cyanobacteria cells can be monitored by following PBS fluorescence quenching in a pulse amplitude modulated (PAM) fluorometer. The three levels of fluorescence that can be measured with this fluorometer (minimal fluorescence [Fo], maximal fluorescence [Fm and Fm′ {in light}] and steady-state fluorescence [Fs]) decrease during exposure of cells to strong blue–green light (Wilson et al., 2006). The complete PAM fluorescence traces and details about the measurements are shown in Supplemental Figure S1. The decrease in maximal fluorescence (Fm′) induced by strong blue light is considerably larger in Δ*apcC* than in WT *Synechocystis* cells (Figure 1, C). Hence, these results were the opposite of those obtained in vitro; in this case, the lack of ApcC had a positive impact on OCP-related photoprotection while in vitro the impact had been negative.

Several experiments were performed to explain the difference in the OCP-related photoprotective PBS fluorescence quenching observed in vitro and in vitro. Because there is often a direct relationship between the OCP concentration and the amplitude of PBS fluorescence quenching (Kirilovsky, 2007), the concentration of OCP was compared in both strains. Immunoblot analysis clearly showed that the OCP concentration was considerably higher in the Δ*apcC* mutant than in the WT (Figure 1, D). We wondered: Why and how would the absence of ApcC induce the increase in OCP concentrations? This effect could be linked to changes in the PSII effective antenna size, which depends on PBS size and on energy transfer from the PBS to the photosystems. Absorbance spectra of WT and Δ*apcC* cells were similar, indicating similar concentrations of chlorophyll and phycobiliproteins in both types of cells and a similar PC to chlorophyll ratio (inset in Figure 1, E). Thus, the PBS size was similar in the WT and the mutant. To investigate energy transfer from the PBS to photosystem II (PSII), the fluorescence induction curves in the presence of DCMU and DBMIB were compared in untreated and in high light-treated cells (Calzadilla et al., 2019). In the presence of DCMU, the rate of fluorescence increase depends only on the antenna size while DBMIB was added to avoid an increase in fluorescence during the measurements due to state transitions. Specific illumination of either photosystem (PSI or PSII) creates an imbalance in photosystem activities leading to formation of dangerous reactive oxygen species. Thus, this needs to be rebalanced via a reorganization of the photosynthetic apparatus via a regulatory mechanism called state transitions. This mechanism, which is accompanied by changes in fluorescence, is induced by oxidation or reduction of the plastoquinone pool generated by the imbalance of photosystem activities. State I transition, accompanied by an increase of PSII fluorescence, is induced by oxidation of the plastoquinone pool during illumination in the presence of DCMU. DBMIB blocks the reoxidation of the reduced PQ pool which keeps the cells in State II, characterized by low PSII fluorescence. Figure 1, E shows the kinetics of fluorescence induction induced by nonsaturating orange light (preferentially absorbed by phycobiliproteins) in dark-adapted and high light exposed WT and Δ*apcC* cells. The absence of ApcC slowed down the kinetics of fluorescence induction in untreated and high-light treated cells, showing a decrease in energy transfer from PBS to PSII in Δ*apcC* cells. The effect was more pronounced in high light-treated cells because, in Δ*apcC* cells, OCP induced a larger fluorescence and energy quenching than in WT cells. In Δ*apcF* and *apcE*-C190S *Synechocystis* mutants, the energy transfer to the photosystems was also perturbed (Calzadilla et al., 2019), but the concentration of OCP did not increase (Supplemental Figure S2). Therefore, we considered the possibility that the enhanced concentration of OCP observed herein was not linked to changes in PSII effective antenna size but instead was caused by an unknown regulatory mechanism directly linked to the *apc* operon.

### An sRNA originates from the 3′-end of the *apc* operon

Based on a partially conserved sequence and secondary structure, an sRNA called SyR2 (for *Synechocystis* ncRNA 2) was previously predicted for the *apcC* 3′-UTR in *Synechocystis* (Voss et al., 2009). We hypothesized that this sRNA could be a possible regulatory element of *ocp* expression. SyR2 was previously demonstrated to accumulate as an individual sRNA of ∼140 nucleotides (nt), likely starting from a position within *apcC* (Voss et al., 2009). In addition, a contiguous, 1,619 nt-long transcriptional unit, TU1472, which encompasses the three protein-coding genes *apcA*, *apcB*, *apcC*, and the 3′-end region of the operon, corresponding to the major part of SyR2, was defined in a study addressing both transcriptional start sites and transcript ends with high sequencing coverage (Kopf et al., 2014).

**Figure 2:**
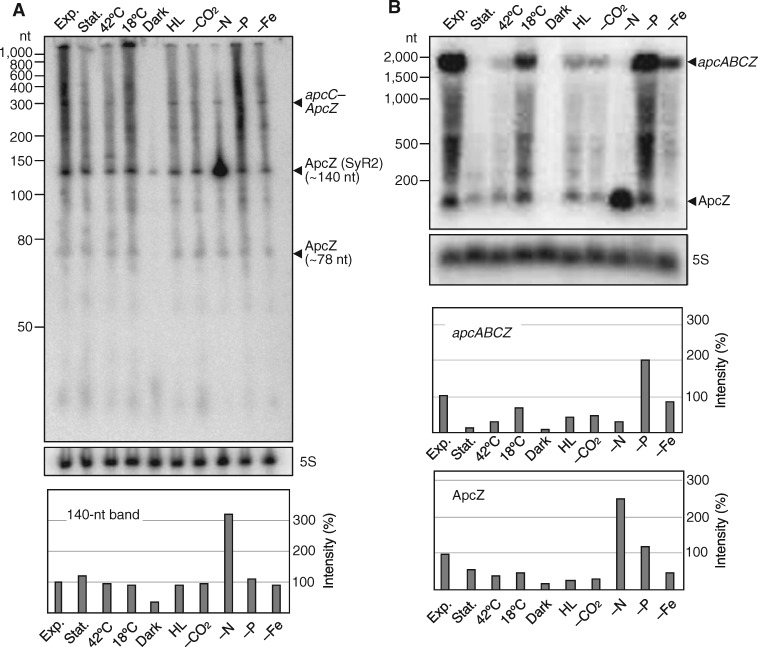
ApcZ belongs to transcripts of different lengths. (A) RNA gel blot of total RNA using a ^32^P-labeled transcript probe specific for ApcZ after separation of 12 µg RNA each isolated from cultures grown under 10 different conditions on a denaturing 10% polyacrylamide gel. (B) RNA gel blot hybridization of the same samples after electrophoretic separation on a 1.5% denaturing agarose gel. The full-length tetracistronic transcript (*apcABCZ*) and ApcZ accumulate differently under different growth conditions. Labels are as follows: exp. (exponential phase), stat. (stationary phase), 42°C (heat stress), 18°C (cold stress), dark (darkness), HL (high light stress), -CO_2_ (limitation in inorganic carbon supply), -N (nitrogen limitation), -P (phosphorus limitation), and -Fe (iron limitation). A hybridization for the 5S rRNA was used for a loading control. Lower panels: The image processing program ImageJ (version 1.52a) was used to compare the density of selected bands after hybridization. Lane profile plots were generated by outlining lanes with the rectangular selection tool. Peaks of interest were enclosed by drawing a base line. The size of this enclosed area was measured by using the wand tool. These arbitrary numbers were used to express the density of each band. 5S values were normalized by dividing each 5S value by the mean value of all 5S bands. ImageJ numbers for each lane were then divided by the normalized 5S value and each value was plotted relative to the exponential phase standard in %.

To confirm the presence and size of this sRNA and to check its regulation, RNA was isolated from WT *Synechocystis* cells exposed to different growth and stress conditions (exponential and stationary growth, exposure to 18°C or 42°C, darkness, high light, depletion of CO_2_, nitrogen, phosphate, or iron; for details, see the “Materials and methods” section). The sRNA was detected in a high-resolution 10% urea polyacrylamide gel by RNA gel blot hybridization with a short, isotope-labeled single-stranded RNA probe against SyR2. A transcript of approximately 140 nt was detected as the major accumulating species (Figure 2, A). Such 3′-UTR-derived sRNAs are commonly named after the mRNA to which they are linked, e.g. SdhX, NarS, or CpxQ for sRNAs originating from the *sdhCDABsucABCD* operon, the *narK* or *cpxP* mRNAs (Chao and Vogel, 2016; De Mets et al., 2019; Wang et al., 2020). Therefore, we renamed this sRNA as ApcZ. The accumulation of ApcZ depended on the growth and stress condition. In addition to the major 140-nt transcript also a less abundant transcript of approximately 78 nt was observed, as well as several longer transcripts encompassing sections of the upstream gene, *apcC.* A transcript of approximately 300 nt was long enough to contain the complete *apcC* coding section together with ApcZ (Figure 2, A).

Using the same probe as in Figure 2, A, both the free ApcZ and the full-length *apcABC*–ApcZ transcript were detectable in a RNA gel blot hybridization following denaturing agarose gel electrophoresis (Figure 2, B). Under most of the chosen stress conditions, both the full-length *apcABC*–ApcZ and the free ApcZ transcript abundances decreased compared with the samples from the exponential growth phase. This decrease in the abundances of both the full-length and the short transcripts were most pronounced in the cells from stationary phase, and signals disappeared almost entirely in the cells incubated in darkness for 12 h. However, phosphate depletion did not induce this decrease, consistent with the rather mild effect of this stress on the transcriptome observed previously (Kopf et al., 2014). Under nitrogen starvation, the full-length transcript disappeared, whereas ApcZ was highly overaccumulated (Figure 2, B). The disappearance of the *apcABC*–ApcZ full-length transcript is consistent with previous studies showing that the expression of phycobiliprotein genes stops when the nitrogen supply becomes scarce (Klotz et al., 2016). The distinctively high concentration of ApcZ indicated that free ApcZ could accumulate separately and to a substantial amount, even when the main part of its operon was repressed. This result suggested the presence of an independent promoter for ApcZ inside the coding sequence of *apcC*, possibly in addition to an instance of operon discoordination and selective stabilization of the ApcZ 140-nt species.

**Figure 3:**
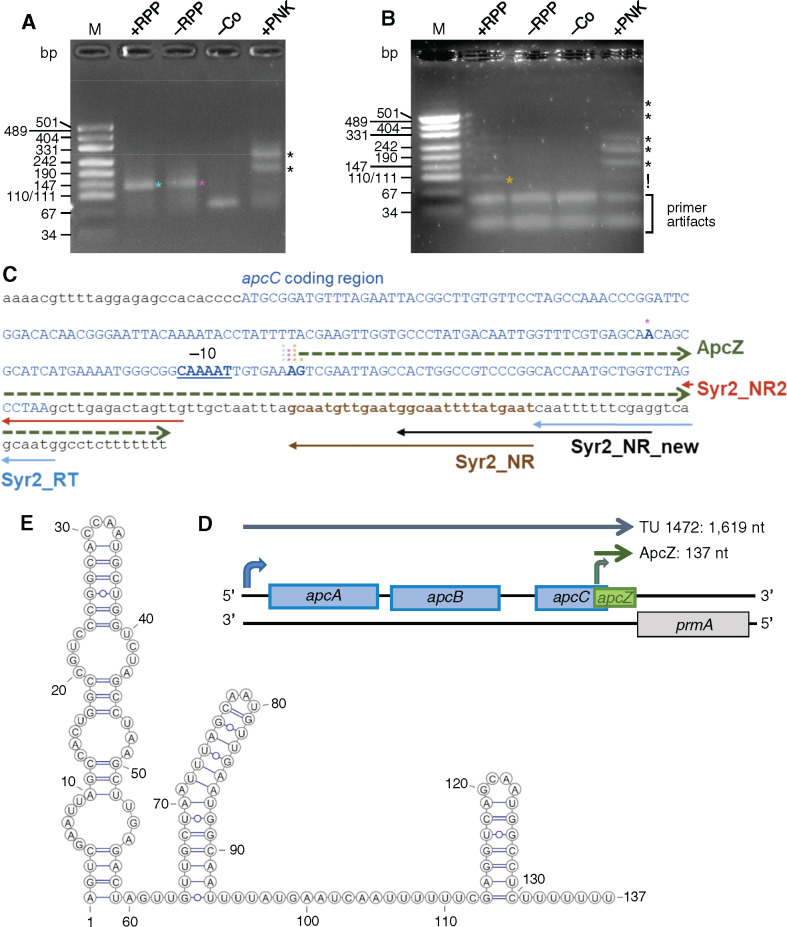
5′-RACE experiments to determine the origin of ApcZ. (A) Amplification products of the 5′-RACE analyses using the specific primer Syr2_NR_new and the adapter primer Adapt52 in the first PCR, followed by a second PCR with nested primers Syr2_NR and Adapt52nest. (B) The 5′-RACE using the primers Syr2_NR2 and Adap52 in the first PCR followed by a second PCR with nested primers Syr2_NR2 and Adapt52nest. Products of low molecular weight also occurring in variant (iv) of the protocol were dismissed as artifacts. In both panels, total RNA from the nitrogen limitation sample in Figure 2 was used. The RNA either was treated with RppH (+RPP), enriching for 5′-ends originating from a transcriptional start site but not selecting against monophosphorylated ends, or was not treated (–RPP), allowing ligation of the RNA linker only to preexisting monophosphorylated 5′-ends. After treatment with PNK, also nonphosphorylated 5′-hydroxyl ends were ligated. –Co, negative control without the 5′-adapter and without any enzymatic treatment of RNA. Specific amplification products are labeled with asterisks. Primer artifacts in panel B are indicated; DNA of plasmid pUC19 digested by *Hpa*II served as size standard. (C) Details regarding the primer locations used in parts (A) and (B; solid arrows) and sequence details inferred from 5′-RACE and 3′-RACE analyses. The *apcC* coding sequence is shown in blue capital letters, the mapped start site of transcription for the major 137-nt form of ApcZ (green dashed arrow) is highlighted by boldface letters and the deduced –10 promoter element is underlined. Asterisks in the same colors as in parts (A) and (B) show the 5′-ends of individual RACE products. (D) Transcription of ApcZ from the 3′-end of the *apcABCZ* operon. The bend green arrow indicates the start site from which the 137-nt major sRNA form originates. The start site for the full-length *apcABC–*ApcZ transcript (TU1472) is also indicated (blue arrow). (E) Secondary structure of the ApcZ major form as predicted by the RNAfold algorithm.

We performed 5′-RACE experiments to further characterize the origin of the different ApcZ forms and to elucidate the first nucleotide of the transcript (Figure 3). Total RNA isolated from the nitrogen starvation sample was split into four parallel reactions and treated in various ways: in the presence (i) and absence (ii) of RNA 5′-pyrophosphohydrolase (RppH); in the presence of polynucleotide kinase (PNK; iii) and finally as control (iv) in which RppH and the RNA oligonucleotide 1, which serves as 5′-linker for ligation, were omitted. After initiation of transcription, the primary RNA 5′-ends in bacteria carry a triphosphate that remains at the first incorporated nucleotide. In order to ligate it to a synthetic RNA adapter facilitating cloning and subsequent sequencing for 5′-RACE, RppH, which converts the triphosphate at primary 5′-ends into a 5′-monophosphate (Almeida et al., 2019) must be added. In the absence of RppH, only the transcripts containing a 5′-monophosphate will be detected. The transcript 5′-monophosphate ends can have two origins: (1) they can originate from cleavage by certain endoribonucleases and (2) the triphosphorylated 5′-ends of primary transcripts can intracellularly (or after RNA extraction) convert to 5′-monophosphates. In transcripts resulting from RNA processing or degradation, RNA 5′-ends carry only a hydroxyl group. To identify such ends, RNA was treated with PNK generating monophosphorylated 5′-ends needed for linker ligation and cloning. In addition, we used different primers to enhance specificity and sensitivity, as indicated in Figure 3, C.

Following reverse transcription and amplification, the four different treatments yielded different band patterns. The presence of a distinct ∼130-nt product following treatment (i) indicated the presence of a primary transcript resulting from initiation of transcription. A similar product was also observed in variant (ii) although a slight smear indicated the likely presence of further transcripts (Figure 3, A, green and pink asterisks). After cloning and sequencing of the amplified product, the 5′-end of ApcZ was assigned to position 1431853 in the chromosome, 46 nt upstream of the *apcC* stop codon (4/4 sequenced clones; Figure 3, C and D). Repetition of the experiment with a primer shifted upstream yielded the same initiation site of transcription (4/5 sequenced clones) and a single-clone mapping the 5′-end to position 1431854, an offset of 1 nt (Figure 3, B and C). Sequence analysis of clones from treatment (ii) yielded 5′-ends mapping to position 1431853 as well (three-fourth clones). Identical 5′-ends were mapped when RNA from exponential growth phase was used (Supplemental Figure S3, A).

After treating the RNA with PNK (iii), several additional bands of higher molecular weight were obtained (Figure 3, A and B, black asterisks). This result indicated that a substantial fraction of transcripts possessed unphosphorylated 5′-ends and likely were derived from processing of the operon RNA. Amplicons indicating the presence of complete *apcC*–ApcZ transcripts resulting from processing of the initial tetracistronic RNA were detected (RACE products larger than 260 [Figure 3, A] and 220 [Figure 3, B] in the +PNK panels).

We also performed 3′-RACE experiments using RNA isolated from cells in the exponential growth phase (see the “Materials and methods” section for details). After cloning and sequencing of the amplified product, the 3′-end was assigned to position 1431989 (3/4 sequenced clones), leaving only a 6-nt spacer toward the end (stop codon) of the *prmA/sll1909* reading frame on the reverse complementary strand (Supplemental Figure S3, B and C). This result was consistent with previous analyses defining the 3′-end of TU1472 (Kopf et al., 2014).

The 5′ and 3′-ends obtained are consistent with a 137-nt sRNA as the major accumulating form of ApcZ. Its predicted secondary structure consists of an elongated stem loop interrupted by three internal loops within the first 59 nt followed by a more elongated second region containing a stem–loop with a single bulging A and a typical Rho-independent terminator structure at the 3′-end consisting of a 7 base pair stem, a 4-nt loop, and an oligo-U run (Figure 3, E).

### ApcZ regulates OCP synthesis

**Figure 4:**
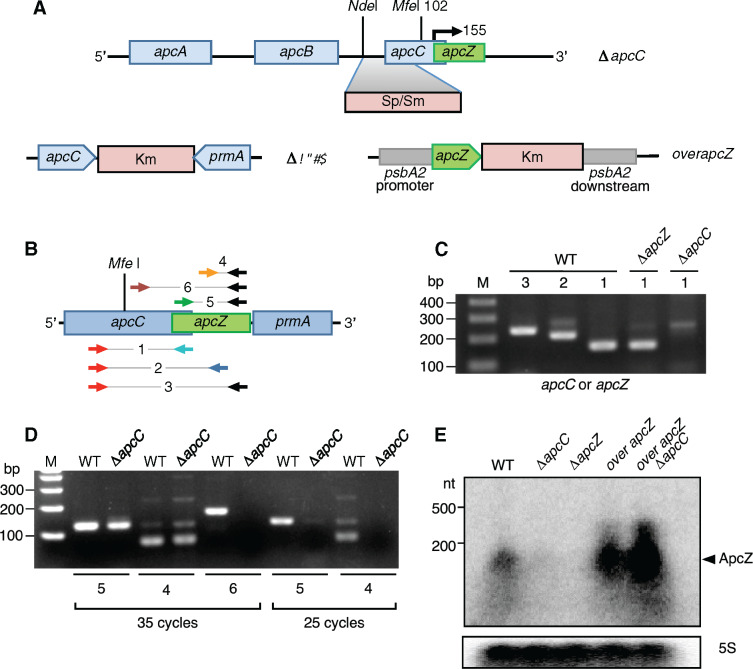
Construction of mutants and detection of *apcC–apcZ* joint transcripts and free ApcZ. (A) In the Δ*apcC* mutant, the insertion of a Sp/Sm resistance cassette replacing the *Nde*I/*Mfe*I restriction fragment by homologous recombination inactivates the *apcC* gene. The start point of ApcZ, as inferred from the results in Figure 3, is indicated by a bent arrow. In the Δ*apcZ* strain, the *apcZ* sequence was replaced with a kanamycin resistance cassette. In the *overapcZ* strains in the WT and Δ*apcC* backgrounds, *apcZ* expression is transcribed from the strong *psbA2* promoter following recombination into the *psbA2* locus. (B) The locations of the oligonucleotide primers used for PCR amplification shown in parts (C) and (D). More details and sequences are given in Supplemental Figure S4 and Supplemental Table S1. (C) Detection of *apcC* mRNAs. Oligonucleotide pair 1 was used to amplify *apcC* alone (1) and pairs 2 and 3 to amplify *apcC* and ApcZ together (2 and 3). 35 PCR cycles were applied. (D) ApcZ detection. Oligonucleotide pairs 4–6 were used to detect ApcZ in WT and Δ*apcC*; 25 PCR cycles (right); 35 PCR cycles (left). All the controls (no RT-PCR) were negative. (E) RNA gel blot analysis of total RNA, isolated from the different mutants, using a ^32^P-labeled transcript probe specific for ApcZ after electrophoretic separation on a 1.5% denaturing agarose gel. Hybridization for the 5S rRNA was used for loading control.

Once the existence of ApcZ was confirmed and its expression characterized under different environmental conditions, we wanted to elucidate whether the increase in OCP synthesis was related to the lack of ApcC or the lack of ApcZ. The Δ*apcC* mutant was previously constructed by replacing part of the intergenic region between *apcB* and *apcC* and the 5′-half of *apcC* with a spectinomycin/streptomycin resistance cassette (Harris et al., 2016; Figure 4, A). In this way, the transcription of the *apc* operon and of ApcZ as part of the tetracistronic operon was interrupted. Moreover, the presence of the cassette may interfere with the normal function of an *apcZ* promoter located within the *apcC* gene because of the distance of only 53 nt between the end of the inserted cassette and the mapped ApcZ start site. Thus, in the Δ*apcC* mutant, ApcZ could also be absent or decreased in abundance.

We constructed three further mutants: one lacking *apcZ*, the other two overexpressing it. To delete *apcZ* but not *apcC*, a kanamycin (Km) resistance cassette was introduced just after the end of the *apcC* coding sequence, thereby replacing nucleotides 50–137 of the *apcZ* sequence corresponding to the *apcC* 3′-UTR, yielding strain Δ*apcZ* (Figure 4, A). To increase the concentration of ApcZ, the entire *apcZ* gene (beginning at the mapped start site of transcription) was introduced into an ectopic site replacing the *psbA2* (*slr1311*) gene in the WT and in Δ*apcC*, bringing *apcZ* under control of the strong *psbA2* promotor to yield strains *overapcZ/WT* and *overapcZ/*Δ*apcC* (Figure 4, A).

Total RNA was isolated from the WT and the Δ*apcC* and Δ*apcZ* mutants, and RT-PCR was performed to detect the presence or absence of transcripts containing *apcC* and/or ApcZ. Six different primer pairs were used to amplify the resulting cDNA (Figure 4, B and Supplemental Figure S4). In the WT, RNAs containing both *apcC* and ApcZ were detected, confirming that ApcZ was indeed transcribed as part of the *apc* operon (Figure 4, C). In Δ*apcZ*, the *apcC* mRNA was detectable while it was absent in Δ*apcC*. In this mutant, ApcZ was detectable when 35 amplification cycles were performed, but not when only 25 PCR cycles were applied, suggesting that its abundance was lowered in Δ*apcC* (Figure 4, D). This was confirmed by RNA gel blot experiments in which ApcZ was clearly present in the WT but almost undetectable in the Δ*apcC* mutant (Figure 4, E). In addition, these experiments showed that the strains overexpressing ApcZ, *overapcZ/*WT and *overapcZ/*Δ*apcC*, contained a largely enhanced amount of ApcZ. Immunoblotting showed that ApcZ was absent in the Δ*apcZ* mutant (Figure 4, E).

Two primer pairs were used to characterize the ApcZ present in Δ*apcC*: one internal and the other amplifying ApcZ from its first nucleotide (+1) defined by the 5′-RACE experiment (Figure 4, B and D). In both cases, clear bands were observed (with 35 cycles) confirming the start point (first nucleotide) of ApcZ. By contrast, when primer pair 6 was used amplifying the last half of *apcC* (after the antibiotic cassette) together with ApcZ, a band was observed in the WT but not in Δ*apcC* (Figure 4, D). These results strongly supported that the free ApcZ present in the Δ*apcC* mutant was synthesized from its own promoter located after the antibiotic cassette.

**Figure 5:**
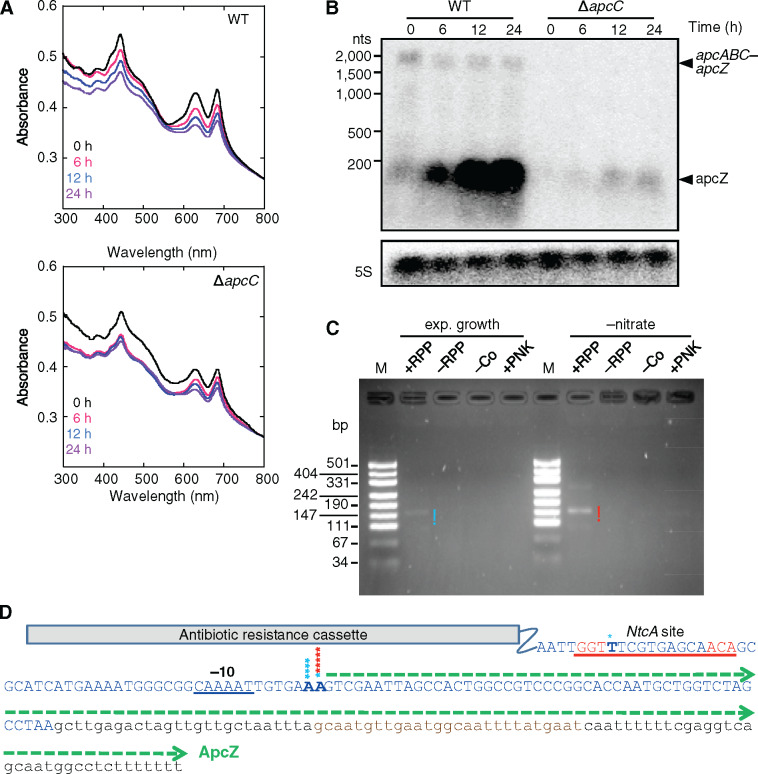
Nitrogen starvation experiment with WT and Δ*apcC* cells. (A) Absorbance spectra of WT and Δ*apcC* cells during nitrate starvation. The 685-nm peak is related to chlorophyll *a* and the 635-nm peak is related to PC. (B) RNA gel blot analysis of total RNA isolated from WT and Δ*apcC* cells grown in the absence of nitrate 0, 6, 12, and 24 h, using a ^32^P-labeled transcript probe specific for ApcZ after electrophoretic separation on a 1.5% denaturing agarose gel. Hybridization for the 5S rRNA was used for loading control. (C) 5′-RACE analysis using RNA isolated from Δ*apcC* cells and the same four parallel reactions and primers as in Figure 3. (D) Sequence details inferred from 5′-RACE in Δ*apcC*. The *apcC* coding sequence is shown in blue capital letters; the position of the antibiotic resistance cassette is marked. Asterisks in the same colors as in panel C above nucleotides in boldface letters show the 5′-ends of individual RACE products. The green dashed arrow indicates the 137-nt ApcZ form beginning with the mapped start site of transcription. The possible NtcA binding site is underlined in red and the –10 element in black.

To test this hypothesis and to further study the regulation of ApcZ expression in Δ*apcC*, we followed the accumulation of ApcZ during nitrate starvation in WT and Δ*apcC* cells. Absorbance spectra showed that the concentration of chlorophyll and phycobiliproteins progressively decreased during the first 24 h of starvation in both strains (Figure 5, A and Supplemental Figure S5). At time 0, in the WT, both free ApcZ and the entire *apcABC–apcZ* mRNA–sRNA were detected. In contrast, both transcripts were undetectable in Δ*apcC* confirming that the concentration of free ApcZ is very low in this mutant. However, a band corresponding to free ApcZ became detectable after 6 h of nitrate starvation and then increased in abundance (Figure 5, B and Supplemental Figure S5). In Δ*apcC*, ApcZ could only be synthesized from its own promoter because the long *apcABC–apcZ* transcript does not exist.

To map the transcriptional start site associated with this promoter unequivocally, the 5′-RACE experiments were repeated with RNA isolated from Δ*apcC* cultures growing exponentially or under nitrogen starvation. Amplified products were obtained only with the RppH-treated RNA fractions, enriching for 5′-ends that originate from a transcriptional start site, but not if RppH was omitted (Figure 5, C). After sequencing, the transcriptional start sites were assigned to nucleotides matching the same two consecutive adenosine residues as in the 5′-RACE analysis of RNA from the WT (Figure 5, D). Moreover, the signal intensity was higher for the nitrogen-starved cells, consistent with the results of the RNA gel blot analysis (Figure 5, B) and indicating that this promoter was upregulated by nitrogen starvation. Furthermore, in WT cells, the concentration of free ApcZ largely increased while the concentration of the long transcript decreased, consistent with the observations in Figure 2, also pointing at the inducibility of this promoter.

Under nitrogen starvation, NtcA, a widespread and highly conserved DNA-binding protein in cyanobacteria, regulates the transcription of not only genes required for nitrogen assimilation but also many other genes (Vega-Palas et al., 1992; Herrero and Flores, 2019). The consensus sequence for the preferred NtcA-binding site within the promotor regions of these genes is often given as GTAN_8_TAC (Giner-Lamia et al., 2017). However, previous work also reported a longer (by 2 nt) recognition sequence TGTN_9/10_ACA (Ramasubramanian et al., 1994) or TGTAN_8_TACA (Jiang et al., 1997, 2000). NtcA can function as a repressor as well as an activator of transcription, depending on the distance between the binding site and the start site of transcription. Binding sites for the activation of transcription are centered at position –41.5 with regard to the transcriptional start (Giner-Lamia et al., 2017). Interestingly, a putative NtcA-binding site GGTTN_8_AACA is centered at position –41.5 with respect to the first nucleotide of ApcZ as detected in our 5′-RACE analyses with RNA from both the WT and Δ*apcC* mutant (Figure 5, D). This suggests that NtcA could be involved in the upregulation of *apcZ* under nitrate starvation. The quantitative difference between the ApcZ level in WT and Δ*apcC* cells was probably related to a perturbation of *apcZ* transcription due to the antibiotic resistance cassette inserted just 4-nt upstream (Figure 5, D). Nevertheless, we cannot discard the possibility that in WT cells, a fraction of the total ApcZ pool resulted from processing of the long transcript and selective stabilization, especially under nitrate starvation conditions.

To confirm that ApcC was present in the PBS from the Δ*apcZ* mutant, the PBSs were isolated and the OCP effect on these PBSs and on those of the WT was compared. OCP induced the same amplitude of PBS fluorescence quenching in both types of PBSs under strong blue light, indicating the presence of ApcC in Δ*apcZ* PBS (Supplemental Figure S6). Moreover, the presence of ApcC was confirmed by mass spectrometry (Supplemental Figure S7). Thus, the replacement of the *apcZ* nucleotides 50–137 (the *apcC* 3′-UTR) by a Km resistance cassette in Δ*apcZ* did not interfere with the expression of ApcC at the RNA or the protein level.

**Figure 6:**
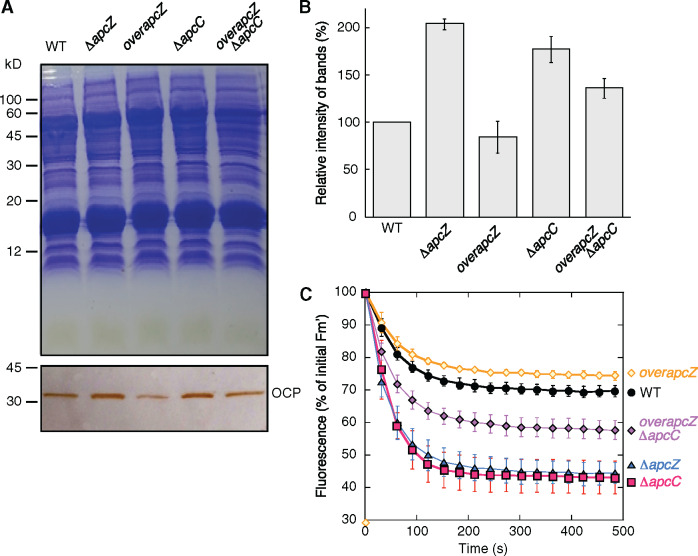
The OCP and the OCP-related NPQ in the different mutants. (A) OCP detection by immunoblot using antibodies against *Synechocystis* OCP in WT and mutant cells. Coomassie Brilliant Blue-stained gel electrophoresis and immunoblot. Equal amounts of proteins were loaded in each lane. For details see the “Materials and methods” section. (B) Comparative densitometry of OCP bands. The results represent the average of three independent experiments. The error bars represent sd. (C) Fluorescence quenching induced by strong blue–green light in WT and mutant *Synechocystis* cells. WT (black circles), single mutants *overapcZ* (orange diamonds), Δ*apcC* (fuchsia squares) and Δ*apcZ* (blue triangles), and the double mutant *overapcZ*/Δ*apcC* (violet diamonds) were illuminated with strong blue–green light (1,200 µmol photons m^−2^ s^−1^). The decrease of Fm′ measured with a PAM fluorometer is shown. The graph shows the mean of three independent biological samples. The error bars represent sd.

We then compared the quantity of OCP and the amplitude of fluorescence quenching induced by strong blue green light in the WT and the four mutants (Δ*apcC*, Δ*apcZ*, *overapcZ*/WT, and *overapcZ/*Δ*apcC*) to determine whether the increased OCP concentration was related to the absence of ApcC or that of ApcZ (Figure 6). The Δ*apc*Z and Δ*apcC* mutants had more OCP than the WT and greater blue–green light-induced fluorescence quenching (60% versus 30%). In the *overapcZ* strains, the concentration of OCP was lower than in the respective recipient strains (WT and Δ*apcC*), and the amplitude of fluorescence quenching induced by strong light was smaller than in the WT and Δ*apcC* (24% versus 30% and 40% versus 60%). These results clearly demonstrated a negative correlation between the presence of ApcZ and the OCP concentration. A higher concentration of ApcZ decreased the OCP concentration, and the absence (or decrease) of ApcZ increased the OCP concentration and as a consequence the amplitude of PBS fluorescence quenching. Thus, ApcZ has an inhibitory effect on OCP expression. We concluded that this effect must be mediated through the part of the *apcZ* sequence that was deleted in strain Δ*apcZ* (nucleotides 50–137), which corresponds to the *apcC* 3′-UTR.

### Characterization of ApcZ as a regulator of *ocp* expression

**Figure 7:**
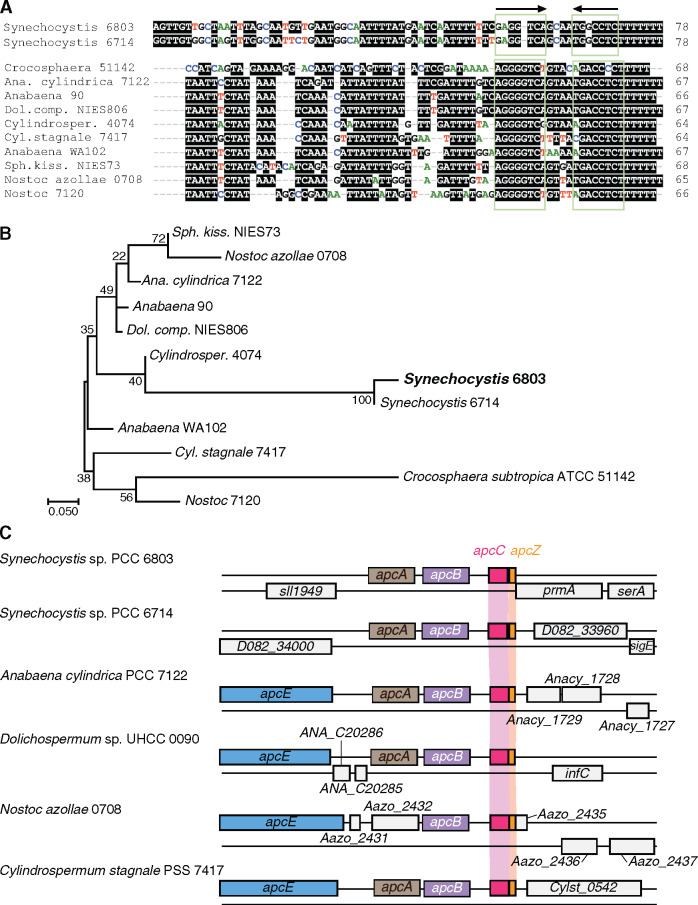
Homologs of ApcZ exist in several cyanobacteria. (A) and (B) Sequence alignment (A) and phylogenetic tree (B) of the same set of ApcZ homologs inferred by maximum likelihood based on the Tamura–Nei model (Tamura and Nei, 1993) as implemented in MEGA7 (Kumar et al, 2016). The tree with the highest log likelihood (–305.51) is shown. The percentage of trees in which the associated taxa clustered together is shown next to the branches if >70. The tree is drawn to scale, with branch lengths measured in the number of substitutions per site. (C) Synteny among *apcZ* loci (red) in two *Synechocystis* strains and four filamentous species at the very 3′-end of the *apcABC* operon, which in the filamentous strains belongs to an even larger gene cluster also encoding the PBS L_CM_ core-membrane linker polypeptide ApcE. The sequences used are from *Synechocystis* strains 6803 (GenBank acc. NC_000911) and 6714 (CP007542), *Cyanothece* sp. ATCC 51142 (NC_010546), *Anabaena cylindrica* PCC 7122 (AP018166), *Anabaena* sp. 90 (NC_019427), *Dolichospermum compactum* NIES-806 (NZ_AP018316), *Cylindrospermum* sp. NIES-4074 (AP018269), *Cylindrospermum stagnale* PCC 7417 (CP003642), *Anabaena* sp. WA102 (CP011456), *Sphaerospermopsis kisseleviana* NIES-73 (AP018314), “*Nostoc azollae*” 0708 (NC_014248) and *Nostoc* sp. PCC 7120 (NC_003272).

The conservation of an sRNA in a wider set of taxa is an indicator of its functional relevance. Here, we focused on the 78-nt stretch following the elongated stem loop in the ApcZ secondary structure (Figure 3, E) and representing the *apcC* 3′-UTR. This was performed to avoid the identification of many homologs due solely to containing the coding region of *apcC*, a conserved gene. Using the GLASSgo algorithm (Lott et al., 2018), putative *apcZ* homologs with a striking similarity in the terminator region were identified in several additional cyanobacteria, including unicellular and filamentous, free-living and at least one symbiotic species (Figure 7, A and B). All these *apcZ* homologs were present in strains containing OCP and originating at the end of the *apc* operon, as in *Synechocystis* (Figure 7, C).

To examine the correlation between the presence of ApcZ and OCP further, we checked the presence of possible *apcZ* homologs in cyanobacteria previously identified as lacking OCP (Kirilovsky and Kerfeld, 2013). Prominent examples of strains without OCP are *Thermosynechococcus elongatus* and other thermophilic *Thermosynechococcus* strains, *Synechococcus elongatus* sp. PCC 7942, *Acaryochloris marina*, all *Prochlorococcus* strains or the symbiontic *Candidatus* Atelocyanobacterium. No *apcZ* candidate sequences were identified in the latter four taxa using BlastN or GLASSgo algorithms with sensitive parameter settings. However, we did find sequences that could constitute candidate homologs in *Synechococcus lividus* PCC 6715, *T. elongatus* PKUAC-SCTE542, *Thermosynechococcus vulcanus* NIES-2134, *Thermosynechococcus* sp. NK55, and *Thermosynechococcus* sp. BP1 (Supplemental Figure S8, A). Two of these strains, *T. vulcanus* NIES-2134 and *Thermosynechococcus* sp. BP1, were cultivated, RNA extracted, and subjected to RNA gel blot hybridization. In contrast to the results obtained for *apcZ* in *Synechocystis*, no evidence for a separate sRNA was obtained. The observed ∼300 nt signal matched the length of the upstream-located gene plus 5′- and 3′-UTR sequences (Supplemental Figure S8, B). Moreover, the *apcZ*-resembling sequences are not linked to the 3′-end of a phycobiliprotein gene but to a gene encoding a CAB/ELIP/HLIP superfamily protein (shown in Supplemental Figure S8, C for *T. elongatus* BP-1). Furthermore, the sequence similarity is pronounced within the region constituting the Rho-independent transcriptional terminator, while the *ocp*-interacting segment according to the analysis in *Synechocystis* (see below) is divergent. We conclude that ApcZ homologs exist in cyanobacteria representing different morphologies and lifestyles, but that it most probably is absent in strains lacking OCP. Hence, a widely conserved function appears to be that is tightly connected to the presence of OCP.

**Figure 8:**
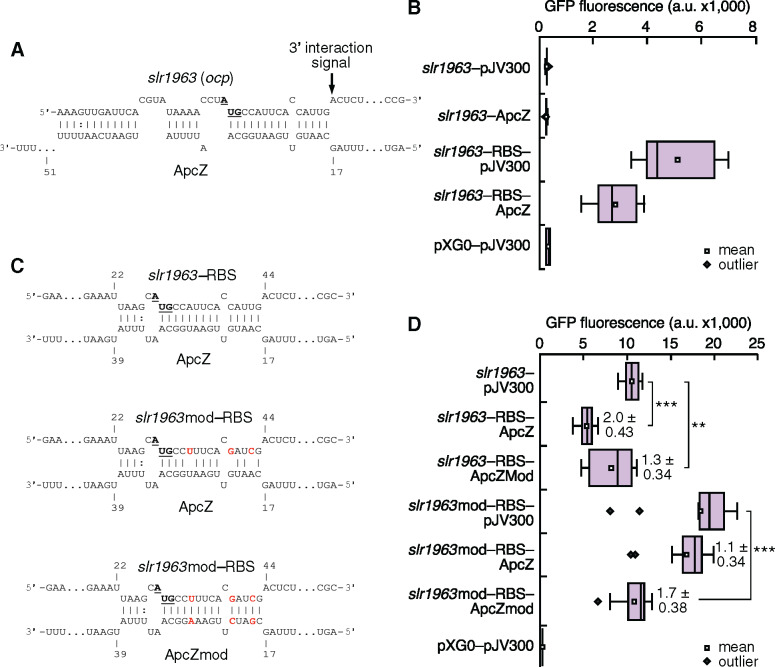
Verification of the direct interaction between ApcZ and the *ocp* mRNA Using an in vivo reporter system. (A) Interaction between ApcZ and the *ocp* mRNA as predicted by the IntaRNA algorithm (Wright et al., 2014). The positions in ApcZ are numbered starting with the first unpaired nt following the elongated stem–loop at position 60 in Figure 3, E, while the transcriptional start site +1 for the *ocp* mRNA was inferred from the previous genome-wide mapping (Kopf et al., 2014). An arrow indicates the adenosine residue at the position of the first noninteracting nt in the *ocp* mRNA, a typical 3′-interaction signal (Papenfort et al., 2010). The *ocp* start codon is highlighted in boldface letters and underlined. (B) GFP fluorescence measurements of *E. coli* TOP10 strains with various combinations of plasmids expressing ApcZ, fusions between the native 5′-UTR-*ocp* (*slr1963*) and *sgfp*, or between the optimized 5′-UTR-*ocp* and *sgfp* (*slr1963*_RBS) are shown. The plasmids pXG-0 (encoding luciferase) and pJV300 (encoding a control RNA) were used as negative controls (for experimental details, see Corcoran et al., 2012). (C) Predicted interaction sites between ApcZ and the *ocp–sgfp* test construct and the respective hybridization energies of the native and mutated sequences at 30°C (the respective substituted nucleotides are boxed in red). The positions in ApcZ and ApcZmod are numbered as in panel A, while the positions in *slr1963*_RBS and *slr1963*mod_RBS are counted from the 5′-end as used in the assay. The *ocp* start codons are shown in boldface letters and underlined. Predictions were made using the IntaRNA webserver (Wright et al., 2014). (D) Repression calculated by dividing the values measured for *slr1963*-RBS-*sgfp* by the corresponding value when ApcZ was present. Autofluorescence measured for negative control cells was subtracted from every measurement before the calculation. The data are presented as the means ± sd from 50,000 individual cells measured for 12 independent colonies. Fold change repression values are given in bold numbers. A one-way analysis of variance (ANOVA) followed by a Bonferroni post hoc test (OriginPro 2020) was used for data analyses (see Supplemental File S1). Significance was established at *P* < 0.00001 = *** and *P* < 0.001 = **.

To predict potential targets of ApcZ, we used the IntaRNA algorithm, considering the folding, hybridization, and conservation of a particular sRNA (Wright et al., 2014). Again, we focused on the 78-nt ApcZ segment beginning with position 60 with regard to Figure 3, E. The results presented in Figure 8 show unequivocally that the ApcZ effect on OCP expression was mediated through this region. Indeed, a high interaction probability was predicted for the *ocp* (*slr1963*) mRNA, placing it at rank 1 (Table 1). The predicted interaction was long with a net interaction energy of –14.4 kcal/mol, encompassing 17 of 24 nt upstream of the *ocp* AUG start codon (including two G:C pairs) and 14 of the 16 following nucleotides with six G:C pairs including the second and third nucleotides of the start codon (Figure 8, A). G:C base pairs with their three hydrogen bonds contribute more to an RNA:RNA interaction than A:U or G:U base pairs with only two hydrogen bonds. Therefore, the majority of the interaction was localized within the first 16 nt of the reading frame rather than within the 5′-UTR. This finding is consistent with the five-codon window hypothesis that posits that an sRNA that base pairs in this region (nucleotides comprising the first five codons of the mRNA) can directly inhibit binding of the 30S ribosomal subunit and thus repress translation initiation, even without sequestering the region upstream of the start codon (Bouvier et al., 2008). Furthermore, we noticed that the first noninteracting nucleotide in the *ocp* mRNA 3′ of the targeted sequence element was an adenosine (Figure 8, A). Such 3′ flanking unpaired adenosine residues have been previously reported as a typical signal for sRNA–mRNA interaction (Papenfort et al., 2010).

**Table 1:** List of the 10 highest-ranking possible mRNA targets of ApcZ (positions 60–137) in *Synechocystis* as predicted by IntaRNA (Wright et al., 2014)

		Target					ApcZ						
ID	Gene	l	Start	End	Seed start	Seed end	Start	End	Seed start	Seed end	Hyb. length	Hyb. energy	Energy
*slr1963*	*ocp*	43	174	216	177	183	18	53	44	50	36	−32.5	−14.4
*slr1813*	*–*	48	57	104	98	104	41	72	41	47	32	−32.7	−14.1
*slr1687*	*nblB2*	40	198^a^	237^a^	225^a^	231^a^	22	68	28	34	47	−32.3	−12.8
*slr1186*	*–*	22	272	293	287	293	47	72	47	53	26	−29.9	−12.6
*sll1272*	*–*	14	78	91	85	91	28	41	28	34	14	−18.7	−12.2
*slr0700*	*potE*	15	141	155	149	155	39	53	39	45	15	−15.5	−11.6
*ssl3441*	*infA*	34	39	72	66	72	41	72	41	47	32	−31.5	−11.5
*sll1968*	*pmgA*	26	70	95	70	76	28	53	47	53	26	−18.4	−11.3
*slr5058*	*–*	18	84	101	93	99	22	41	24	30	20	−19.0	−11.3
*slr2141*	*metF*	16	118	133	120	126	53	67	59	65	15	−25.1	−11.2

In this prediction, 200-nt upstream and 100-nt downstream of every annotated protein-coding gene were considered (start codons at positions 201–203). The columns show the systematic gene ID, followed by the classical gene name if available, the positions, lengths (l), and seed regions of predicted interactions in the target and in ApcZ and the calculated hybridization energies in kcal/mol. For ApcZ, a position of 1 would refer to nt 60 in Figure 3, E. The resulting predictions were ranked by the net energy score with the lowest (best) energy ranking for position 1 (last column).

a These positions were manually corrected by 30 nt to the second in-frame start codon because the annotated start of the coding sequence is outside the transcribed region.

To examine whether the direct ApcZ:*ocp* interaction caused the observed changes in expression, OCP abundance, and fluorescence, the *ocp* 5′-UTR was fused to the gene for the superfolder green fluorescent protein (*sgfp*) and coexpressed with ApcZ in *Escherichia coli*. Consistent with reports on the unique Shine–Dalgarno sequences of cyanobacteria (Wei and Xia, 2019), the native *ocp* 5′-UTR contained no Shine–Dalgarno sequence suitable for *E. coli* and did not yield fluorescence when tested (Figure 8, B). Therefore, it was replaced with the compatible 5′-UTR from plasmid pQE70, directly fused to the start codon of *ocp/slr1963*. The replacement, called *slr1963*-RBS, retained the predicted interacting segment encompassing the AUG and following nucleotides with six G:C pairs and added three possible novel A:U and one G:U pairings upstream of the AUG, which could, however, lead to a less stable interaction than in the unmodified version (compare to the interaction shown in Figure 8, A).

GFP fluorescence was measured in strains carrying various combinations of plasmids (Corcoran et al., 2012). Compared with the control (pXG-0 + pJV300), the strain carrying the *slr1963*-RBS*-sgfp* fusion showed significant GFP fluorescence, demonstrating that the translation initiation from *slr1963*-RBS was functional in *E. coli* (Figure 8, B). In the presence of the ApcZ-expressing plasmid, the GFP fluorescence decreased approximately two-fold, indicating a direct and significant interaction between ApcZ and the *ocp* mRNA (Figure 8, B).

To verify the interaction at the predicted site, point mutations were introduced in the *ocp* mRNA (pos. +6, +12, and +15: with +1 = the A of the AUG start codon) or ApcZ (pos. +19, +22, and +28, Figure 8, C). Indeed, mutation of either one of these sequences diminished the interaction, as indicated by reduced repression (Figure 8, D). However, the combination of both mutations, which are complementary to each other, restored significant repression (Figure 8, D). The measured repression in *E. coli* appeared lower than in *Synechocystis*, possibly because RNA-binding proteins might be involved in the interaction in the cyanobacterium that could not be fully functionally replaced by the enterobacterial RNA chaperones. Nevertheless, these data confirmed the direct interaction of ApcZ with the *ocp*/*slr1963* mRNA, which should also affect the translation of the OCP protein in *Synechocystis*.

## Discussion

Our knowledge of the regulation of *ocp* expression is based on transcriptomic and proteomic studies performed under different stress conditions. These studies suggested a relationship between the redox state of the photosynthetic electron transport chain and the level of *ocp* transcription. Although specific elements of this regulation remain unknown, it is possible that more general histidine kinase-response regulator pairs such as Hik33–RpaB (Wilde and Hihara, 2016; Riediger et al., 2019) and/or specific redox-active transcriptional regulators such as PedR (Nakamura and Hihara, 2006; Horiuchi et al., 2010)) are involved. Here, we describe how we discovered a factor involved in the posttranscriptional regulation of *ocp* expression by characterizing the *Synechocystis* mutant lacking the PBS-core linker ApcC. This factor is ApcZ, an sRNA previously called SyR2 (Voss et al., 2009) originating from the 3′-end of the *apcABC* operon.

The majority of sRNAs in bacteria are transcribed from free-standing genes (for reviews, see Kopf and Hess, 2015; Adams and Storz, 2020). However, a growing number of examples have been found in which an sRNA derived from a polycistronic mRNA regulates another mRNA, leading to the concept of competing endogenous RNAs (Grull and Masse, 2019). In some instances, sRNAs have been described that originate from the mRNA 3′-ends and are involved in different regulatory and physiological pathways (Chao et al., 2012; Eisenhardt et al., 2018). The majority of characterized 3′-end-derived sRNAs were, so far, only described in two bacteria, *E. coli* and *Salmonella typhimurium* (Miyakoshi et al., 2015). ApcZ is the first sRNA of this type that is discovered in cyanobacteria.

Two general types of 3′-end-derived sRNAs have been described in the literature. One type is transcribed from an ORF-internal promoter hidden within the 3′-end of a protein-coding gene on the same strand but shares the transcription terminator with the mRNA, a situation described first for the *Salmonella* sRNA DapZ (Chao et al., 2012). The second type of 3′-end-derived sRNAs is generated by 3′-end cleavage of mRNAs and often comprises just the 3′-UTR, exemplified by the sRNAs CpxQ (Chao and Vogel, 2016) and RaiZ (Smirnov et al., 2017). The existence of an apcC-internal promoter indicates that ApcZ belongs to the first type. However, the reduced amount of ApcZ detected in the ΔapcC mutant (Figures 4, 5) suggests that processing of ApcZ out of the long mRNA could contribute as well, although the insertion of the antibiotic resistance cassette into the first half of apcC might also have perturbed apcZ transcription.

**Figure 9:**
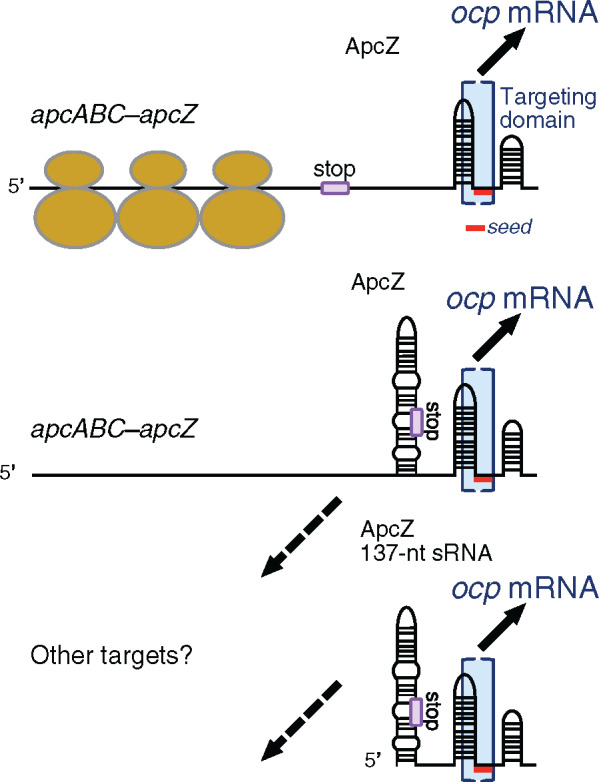
ApcZ is a posttranscriptional regulator. We propose that free ApcZ as well as the entire *apcABC–ApcZ* transcript can interact with the *ocp* mRNA because the targeting domain (blue brackets) is located within the 3′-UTR of the *apcABC–apcZ* transcript. Therefore, it is also available for interaction if the coding sequences are actively translated and covered by ribosomes. Exponential growth is a representative condition for this mode of action (upper panel). If translation ceases but the mRNA–sRNA hybrid transcript or its degradation products remain in the cell, *ocp* mRNA could still be targeted and its translation repressed (middle panel). ApcZ also exists as a separate, free 137-nt sRNA (lower panel). This is the major fraction during nitrogen starvation and a minor fraction under other conditions (compare to Figure 2 and Figure 5, B). Therefore, all three types of ApcZ or ApcZ-containing transcripts need to be low or not transcribed to allow *ocp* translation, which happens after shifts to very high light intensities. Only in the scenario in the middle and at the bottom can the sequence region encompassing the first ∼50 nt of ApcZ interact with other hypothetical targets (dashed arrows). Part of the targeting domain is predicted to be single-stranded and to contain the seed region for interaction (short red line), i.e. a segment capable of forming contiguous base-pairing. This seed region was predicted by IntaRNA (Wright et al., 2014) to extend from position 44 to 50 in the investigated segment (Table 1) corresponding to positions 104–110 in Figure 3, E. The end of the *apcC* reading frame is indicated by a box and the word stop.

It is the presence of ApcZ that is responsible for the repression of ocp translation under nonstressed conditions. Its absence largely increases the OCP concentration. We propose that free ApcZ, the entire apcABC–apcZ mRNA and possibly also shorter transcript turnover products can interact with the ocp mRNA (Figure 9). Although most of the tetracistronic mRNA will be covered by ribosomes during the synthesis of ApcC subunits, the 3′-end of the mRNA will be free, allowing its interaction with ocp mRNA (Figure 9).

**Figure:**
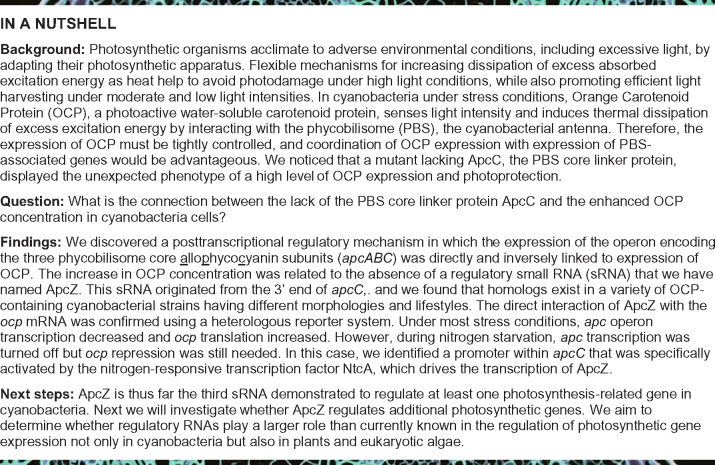


Thus, ApcZ connects the expression of the operon encoding APC subunits of PBS directly and, in an inverse fashion, to the expression of OCP. This inverse connection is important to allow maximal light harvesting under nonstressed conditions and maximal photoprotection under high light or other stress conditions, including low CO_2_ or nutrient starvation. Under these conditions, in which it is important that less energy arrives at the photochemical centers to avoid ROS production, a high OCP-to-PBS ratio is essential for photoprotection. Conversely, under nonstressed conditions, a high concentration of OCP must be avoided to allow maximal photosynthetic capacity. In this latter case, high expression of the apc operon leads to a high concentration of ApcZ, inhibiting OCP synthesis. Under most stress conditions, the expression of phycobiliproteins and linkers decreases. Thus, the apc operon expression also decreases and, as a consequence, the concentration of ApcZ, leading to a higher OCP concentration and a higher OCP-to-PBS ratio. This is important in the first minutes and hours of stress because, in most cases, the PBSs are not actively degraded and their concentration remains high for a relative long period. Hence, this mechanism presents a simple but elegant way to coordinate OCP and antenna gene expression.

In most stress conditions, expression of both the entire operon and ApcZ decreased allowing a larger increase of ocp translation. However, in nitrogen starvation, we identified one exception in which free ApcZ accumulated and the full-length apcABC–ApcZ mRNA almost entirely disappeared. This observation suggests a possible explanation for ApcZ needing its own promoter that could be regulated differently from the apcABC operon. After several hours of nitrogen starvation, transcription of the apc operon is diminished and the PBSs are actively degraded, yet OCP is also not needed. Hence, the transcription of ApcZ from its own promoter is increased, just as we observed in the time-course analysis of the response to nitrogen starvation in WT and ΔapcC cells (Figure 5). In addition, ApcZ appears to become selectively stabilized when the protein-coding part of the apcABC–apcZ mRNA becomes degraded during this condition (Figure 5). This possibility is further supported by the inverse correlation between ApcZ and ocp mRNA observed in response to long-term nitrogen starvation (Klotz et al., 2016), suggesting that ApcZ is integrated into the genetically determined program leading to dormancy under such conditions.

In recent years, hundreds of potentially regulatory sRNAs were identified in different model cyanobacteria including *Synechocystis* (Kopf and Hess, 2015). The majority of these sRNAs are transcribed from their own and often free-standing genes, and they exhibit only short and imperfect reverse complementarity to their target mRNAs. These sRNAs frequently regulate the stability and/or translation of more than one target by forming partial RNA–RNA duplexes. ApcZ seems to belong to this group of sRNAs. Bioinformatics analysis predicted that the part of ApcZ investigated herein could interact with additional mRNA targets including nblB2 and pmgA (Table 1), which were demonstrated to play roles both in the turnover of PBSs (Dolganov and Grossman, 1999) and in photosystem stoichiometry by inducing suppression of PSI and Chl under high light conditions, respectively (Hihara et al., 1998). The regulation of these genes could be similar to that of ocp (Figure 9). In addition, we cannot rule out that the first part of ApcZ could also have regulatory functions. In the tetracistronic mRNA, these first 49 nt of ApcZ coincide with the final codons of the apcC coding region and therefore will be covered by ribosomes most of the time (Figure 9). This coverage will hinder the interaction with other mRNAs. Therefore, such interactions are more realistic only for the free ApcZ form. While these ideas point to a potentially more complex scenario, it is currently entirely hypothetical and remains for future studies.

Therefore, comparing the overall function of ApcZ in context with other 3′-end derived sRNAs, it appears not as a global regulator like CpxQ controlling inner membrane stress (Chao and Vogel, 2016; Grabowicz et al., 2016) or SdhX linking the TCA cycle with other central metabolic pathways (De Mets et al., 2019; Miyakoshi et al., 2019). Instead, ApcZ appears more as a specific posttranscriptional regulator to prevent physiological conflict, here between light harvesting by the PBS and protection from light by OCP. In this sense, ApcZ resembles the functions of several other sRNAs which act as switches or to optimize metabolic activity, as in the case of the s-SodF RNA mediating the inverse expression of the sodF and sodN genes (Kim et al., 2014) or NarS mediating the cross-regulation of nitrate and nitrite transporter genes (Wang et al., 2020).

ApcZ is, in addition to PsrR1 and IsaR1 (Georg et al., 2014, 2017), the third characterized sRNA in *Synechocystis* that functions in the control of photosynthetic gene expression. In addition to these more versatile sRNAs, several cis-antisense RNAs (asRNAs) with a role in photosynthetic gene expression have been described in *Synechocystis*. IsrR regulates the amount of IsiA via codegradation with the isiA mRNA in a threshold linear-response mechanism (Duhring et al., 2006; Legewie et al., 2008; Georg and Hess, 2011). As1_flv4, via a similar mechanism, downregulates the flv2-4 operon (Eisenhut et al., 2012), while RblR, PsbA2R, and PsbA3R play protective roles for their respective partners, the rbcL, psbA2 and psbA3 mRNAs (Sakurai et al., 2012; Hu et al., 2017).

ApcZ appears in several ways unique compared with these previously characterized sRNAs and asRNAs. The present work shows that an sRNA appended to a long operon mRNA with a central function in photosynthetic light harvesting can function as an active regulator in the regulatory network of cyanobacteria. It has been suggested that 3′-UTRs of mRNAs serve as a reservoir for the evolution of new regulatory sRNAs (Miyakoshi et al., 2015; Updegrove et al., 2015). However, the underlying evidence stems mainly from observations in *E. coli* and *S. typhimurium* (Chao and Vogel, 2016; Grabowicz et al., 2016; Holmqvist and Vogel, 2018; De Mets et al., 2019; Miyakoshi et al., 2019; Wang et al., 2020). Our findings that ApcZ, a 3′-UTR-derived sRNA, is a regulator of OCP expression extend the suite of such known sRNAs to the cyanobacteria, which are phylogenetically quite distant from the gammaproteobacteria. They therefore significantly support the idea of mRNA 3′-UTRs as an evolutionary source of novel sRNAs.

## Materials and methods

### Strains and growth conditions

WT and mutant *Synechocystis* cultures were grown at 33°C in BG11 medium (Herdman et al., 1973). Fluorescent white tubes (Gro-Lux, Sylvania) giving an intensity of 50 μmol photons m^−2^ s^−1^ was used for illumination. Cultures were grown on a rotary shaker (120 rpm) under a CO_2_-enriched atmosphere and kept in the logarithmic stage of growth. The Δ*apcC*, Δ*apcZ*, *overapcZ*WT, and *overapcZ*/Δ*apcC* strains were grown in the presence of spectinomycin (20 µg/mL) and/or kanamycin (40 µg/mL). For the RNA gel blots shown in Figure 2, liquid precultures were grown in volumes of 75 mL of BG11 medium in 100-mL Erlenmeyer flasks at 30°C under continuous white light illumination of 50–80 μmol photons m^−2^ s^−1^ and a continuous stream of air to the desired OD_750_ of 0.6–0.8 (exponentially growing cultures). From these, aliquots were exposed to cold stress (15°C for 30 min), heat stress (42°C for 30 min), carbon limitation (cells were washed three times with carbon-free BG11 medium and cultivated for an additional 20 h), darkness (no light for 12 h), Fe^2+^ limitation (by adding the iron-specific chelator desferrioxamine B and cultivating for an additional 24 h), high light (470 μmol photons m^−2^ s^−1^ for 30 min), nitrogen depletion (cells were washed three times with nitrogen-free BG11 medium and cultivated for an additional 12 h), phosphate depletion (three washes with phosphate-free BG11 medium and incubation for another 12 h), and stationary phase (cells were grown to an OD_750_ of 4.7). For the RNA gel blots shown in Figure 5, 300-mL cultures were grown in 1-L Erlenmeyer flasks at 50–80 μmol photons m^−2^ s^−1^ and bubbled with air to an OD_750_ of 0.6–0.8. Cultures were centrifuged and washed three times with nitrogen-free BG11 and set to an OD_750_ at 0.4. Samples were then taken after incubation in nitrogen-free medium for 0, 6, 12, and 24 h with the same light condition and air bubbling.

### Construction of mutants

The construction of the Δ*apcC* mutant has been previously described (Harris et al., 2016). To construct the ApcZ-free mutant Δ*apcZ*, the intergenic region between the genes *apcC* and *prmA* was deleted while the *apcC* coding sequence was kept intact. In this process, a 500-bp fragment upstream and another one downstream of this region were amplified by PCR using genomic DNA of *Synechocystis* as template and ΔSyR2-up-F, ΔSyr2-up-R, ΔSyR2-down-F, and ΔSyR2-down-R. For cloning, *Bam*HI, *Eco*RV, or *Kpn*I restriction sites were included in the synthetic oligonucleotides used for DNA amplification (the sequences of all oligonucleotide primers are provided in Supplemental Table S1). The resulting upstream fragment was digested with *Bam*HI and *Eco*RV and then cloned into the pBluescript SK+ plasmid (Stratagene). The plasmid obtained was then ligated with the *Kpn*I-digested downstream fragment. Colonies containing the inserted downstream fragment in the correct orientation were selected. Finally, the obtained plasmid was digested with *Sal*I and a 1.3-kb kanamycin cassette was inserted. Upon transformation of *Synechocystis* (Grigorieva and Shestakov, 1982) with this plasmid and homologous recombination, the antibiotic cassette replaced the intergenic region.

To construct the ApcZ overexpression mutant (*overapcZ*), *apcZ* was amplified by PCR with primers SyR2-F/SyR2-R containing *Nde*I and *Bam*HI restriction sites, respectively (Supplemental Table S1). The resulting PCR product was digested and ligated into plasmid pPSBA2KS (Lagarde et al., 2000), and a kanamycin resistance cassette was inserted into the *Bam*H1 restriction site. Following transformation into WT and Δ*apcC Synechocystis* strains, this construct recombined into the *psbA2* locus, yielding *apcZ* overexpression under the control of the *psbA2* promoter.

### Total RNA isolation and RT-PCR

Total RNA was isolated from 100 mL of WT and mutant *Synechocystis* cells at OD_800_ = 0.8 using PGTX buffer (Pinto et al., 2009) and treated with RNase-free DNase I (NEB). The PGTX solution has the following composition (for a final volume of 100 mL at pH 4.2): phenol (39.6 g), glycerol (6.9 mL), 8-hydroxyquinoline (0.1 g), EDTA (0.58 g), sodium acetate (0.8 g), guanidine thiocyanate (9.5 g), guanidine hydrochloride (4.6 g), and Triton X-100 (2 mL), final pH approximately 4.2. RT-PCR was carried out using the Prime Script RT reagent Kit (Takara). The reverse primers apcC-R1, Apc-R2, and apc-R3 were used for cDNA syntheses. Then, the cDNAs were amplified by PCR using the oligonucleotides described in Supplemental Table S1.

### 5′- and 3′-RACE experiments and northern blot

For 3′-RACE, total RNA was digested with DNase I (NEB) for 30 min at 37°C. The reactions were stopped by phenol–chloroform extraction followed by ethanol precipitation. Precipitated RNAs were redissolved in DEPC-treated H_2_O. The 3′-RACE assay was carried out essentially as described (Hu et al., 2017) with some modifications. First, RNA was ligated with the 3′-linker (150 pmol) using T4 RNA ligase (NEB; overnight incubation) at 17°C. Then, after a second organic reagent extraction, the 3′ linker-ligated RNA was reverse-transcribed with 100 pmol of 3′ linkerPCRrev using the Prime Script RT reagent Kit (Takara) according to the manufacturer’s protocol. PCR amplification using 3′-RACE-1 or 3′-RACE-2 and 3′ linkerPCRrev primer was conducted with the obtained cDNA as template. Finally, the obtained PCR bands were excised and cloned into a pMD18-T vector (#6011, TAKARA, China). After transformation, colonies were screened by PCR and sequenced to determine the 3′-end. All oligonucleotides and primers used for RACE analysis are listed in Supplemental Table S1.

For RNA gel blot and 5′-RACE analyses, total RNA was isolated as described (Hein et al., 2013) with the modification that the cell material was collected by rapid filtration on hydrophilic polyethersulfone filters (Pall Supor 800 Filter, 0.8-µm pore size) as introduced previously (Kopf et al., 2014). Residual DNA was removed using TURBO DNase (Life Technologies GmbH, USA) in two consecutive steps, in each of which 4 units of DNase were added to 7 µg of total nucleic acids and kept at 37°C for 15 min. Transcriptional start sites were determined by 5′-RACE as described (Kopf et al., 2014), except that treatment with terminator 5′-phosphate-dependent exonuclease was omitted and tobacco acid pyrophosphatase was replaced with RNA 5′-pyrophosphohydrolase (RppH). Briefly, to remove triphosphates from 5′-ends resulting in monophosphate ends, RNA was treated with RppH (7.5 U per reaction; NEB, USA) for 30 min at 37°C. To determine the unphosphorylated processing sites, RNA was incubated for 30 min at 37°C with T4 PNK (15 U per reaction; NEB, USA), which phosphorylates 5′-ends and enables subsequent ligation. Per reaction, 1 µL of RNA oligonucleotide 1 (Supplemental Table S1, concentration 10 µM; Invitrogen, Germany) was ligated to the treated RNA samples using T4 RNA ligase (40 U per reaction; NEB, USA) for 1 h at 37°C. After each enzymatic treatment, the samples were purified with the RNA Clean & Concentrator-5 Kit according to the manufacturer’s instructions (Zymo Research Corporation, USA). Two control reactions were included: in one, RppH was omitted to allow the mapping of preexisting monophosphorylated 5′-ends, and in the other, RppH and RNA oligonucleotide 1 were omitted as a negative control. For reverse transcription, the linked RNA was incubated with 4 U of the Omniscript reverse transcriptase (Qiagen, Germany) in the provided reaction buffer containing 0.08 µM of the gene-specific primer Syr2_RT and 1 mM dNTPs. Incubation was carried out at 42°C for 2 h with a final inactivation step at 95°C for 5 min. All reactions were performed in the presence of 40 U Ribolock RNase Inhibitor (Fermentas, Germany).

The cDNA was amplified by two subsequent PCRs. In the first PCR, the gene-specific primer Syr2_NR_new or Syr2_NR2 (0.2 µM) and the RNA oligonucleotide 1-specific primer Adapt52 (0.2 µM) were used with the following cycling conditions: 94°C/30 s; 32 cycles of 94°C/15 s; 50°C/15; 68°C/30 s; 68°C/5 min in OneTaq reaction buffer containing 1.25 U OneTaq polymerase (NEB, USA), 0.2 mM dNTPs, and 1.8 mM MgCl_2_. The products were separated on 3% Nusieve agarose TAE gels, and the bands of interest were excised and purified on Nucleospin columns (Macherey and Nagel, Germany). The eluates served as template for the second PCR, in which the same gene-specific primers (Syr2_NR2) or the nested gene primer (Syr2_NR) were combined with the nested RNA oligonucleotide 1-specific primer Adapt52nest and the same PCR protocol applied. A complete list of all primers used is provided in Supplemental Table S1. Amplified PCR fragments were gel-excised, purified on Nucleospin columns, and cloned into plasmid pGEMT (Promega, Germany). After transformation into *E. coli* DH5α (Dower et al., 1988), plasmid inserts were amplified by colony PCR (primers CP1 and CP2), purified with ExoSAP-IT (ThermoFisher Scientific, USA), and sequenced at Eurofins, Germany.

For RNA gel blots, RNA samples (5–12 µg) were denatured for 10 min at 65°C in RNA loading buffer (ThermoFisher Scientific). Denatured RNA samples were separated on 10% urea polyacrylamide gels for 16 h at 100 V or on 1.5% denaturing formaldehyde-agarose gels for 1 h at 100 V. The separated RNA on the gels were then transferred to Hybond-N nylon membranes (Millipore, USA) by electroblotting for 1 h at 400 mA or upward capillary transfer overnight. After prehybridization in 50% deionized formamide, 7% SDS, 250 mM NaCl, and 120 mM Na(PO_4_) pH 7.2, the membranes were hybridized with specific [γ-^32^P] ATP end-labeled oligonucleotides or [α-^32^P] UTP-incorporated transcripts. Specific oligonucleotide end labeling was performed with 0.5 U T4 PNK (NEB, USA), 1.25 mM oligonucleotide, and 15 µCi [γ-^32^P] ATP in reaction buffer for 30 min at 37°C. The MAXIscript Kit (Thermo Fisher Scientific) was used for the generation of [α-^32^P] UTP-incorporated transcript probes. Hybridization was performed overnight at 42°C or at 62°C with labeled oligonucleotide probes or labeled transcript probes, respectively. The membranes were washed in 2×SSC (3 M NaCl, 0.3 M sodium citrate, pH 7.0), 1% SDS for 10 min; 1×SSC, 0.5%SDS for 10 min; and briefly in 0.1×SSC, 0.1% SDS. All wash steps were performed 5°C below hybridization temperature. Signals were detected and analyzed on a Storm 820 System (GE healthcare, USA) with Quantity Qualification software.

### Purification of PBSs and OCP

The purification of PBSs was performed according to a procedure derived from the literature (Ajlani et al., 1995). Briefly, harvested cells were washed twice with 0.8 M potassium phosphate buffer (pH 7.5) and resuspended in potassium buffer at a chlorophyll concentration of 1 mg/mL. Cells were then broken by vortexing in the presence of glass beads, 1 mM EDTA, 1 mM caproic acid, 1 mM phenylmethylsulfonyl fluoride, 1 mM benzamidine, and 50 μg/mL DNase. After 2 h of incubation with Triton X-100 (2% v/v), solubilized membrane components were removed by centrifugation at 20,000 × *g* for 20 min at 23°C. The supernatant was loaded onto a 0.25-, 0.5-, 0.75-, and 1.5-M sucrose gradient and centrifuged at 23°C for 12 h. The lower dark blue layer was collected and its absorbance spectrum was recorded. OCP protein was purified from *E. coli* using plasmid Syn-3aaNtag-ECN according to a previously described method (Bourcier de Carbon et al., 2015).

### LC–MS/MS analysis

To confirm the presence of ApcC in the PBSs of the Δ*apcZ* mutant, LC–MS/MS analysis was performed. The isolated PBSs of WT and Δ*apcZ* were precipitated with a final concentration of trichloroacetic acid solution at 10% (v/v). The pellet was washed two times with water before it was resuspended in 172 mM Tris–HCl, pH 8.0. The protein solution obtained was mixed with 3× loading buffer (18% SDS, 1 M sucrose, 0.75% bromophenol blue, 0.58 M Tris–HCl pH 8), and then boiled for 10 min at 95°C. The sample obtained was loaded on a 15% polyacrylamide SDS gel for electrophoresis, and the protein strip from 5 to 15 kD was cut off for enzymatic digestion. Then, the peptides were analyzed by LTQ-Orbitrap Velos for the identification of ApcC protein in the samples. Measurements were performed by the SICaPS service of the Institut de Biologie Intégrative de la Cellule (I2BC), Gif-sur-Yvette, France.

### Immunoblot analysis

Total protein extracts for the immunoblot shown in Figure 6 were prepared as follows: cell pellets were resuspended in 500 µL of 50 mM Tris–HCl (pH 6.8) with protease inhibitors (1 mM caproic acid, 1 mM phenylmethylsulfonyl fluoride, and 1 mM benzamidine; Sigma); approximately 500 µL of glass beads were added, and the cells were broken by five cycles of vortexing for 1 min each, followed by freezing in liquid nitrogen and thawing. Finally, unbroken cells and cell debris were removed by centrifugation (5 min, 6,000 × *g*), and the supernatant was recovered. To measure the protein concentration, Bio-Rad’s protein assay kit was used according to the manufacturer’s instructions. For immunoblot analysis, an equal amount of proteins was loaded per slot and the proteins were separated in a 15% polyacrylamide/2-M urea SDS gel.

For the immunoblots shown in Figure 1 and Supplemental Figure S2, PBS-membrane complexes were prepared as previously described (Wilson et al., 2006). For immunoblot analysis, an equal concentration of chlorophyll (2 µg chl per slot) was loaded and the proteins were separated in 12% polyacrylamide/2-M urea SDS gels. The OCP protein was detected by a polyclonal antibody against OCP used at a dilution of 1:3,000. Anti-OCP polyclonal rabbit antiserum was made (CoVance) using recombinant *Synechocystis* OCP (1.4 mg/mL in 20 mM Tris, pH 8.0, and 30% sucrose). Anti-OCP was purified from the sera using Affi-Gel 15 (Bio-Rad) following the manufacturer’s instructions (Wilson et al., 2007). Binding of the OCP antibody was monitored by an alkaline phosphatase colorimetric reaction.

### Fluorescence measurements

#### PAM fluorometry to follow the decrease in PBS fluorescence

Fluorescence quenching was monitored using a PAM fluorimeter (101/102/103-PAM; Walz) in a 1 × 1-cm square stirred cuvette. Experiments with whole cells were carried out at a chlorophyll concentration of 2.5 μg/mL at 31°C. Dark-adapted cells were first illuminated with weak blue–green light (85 µmol photons m^−2^ s^−1^, Halogen white light filtered by a Corion cut-off 550-nm filter; 400–550 nm) to induce the State I transition, and then the blue-adapted cells were illuminated with strong intensities of the same blue–green light (1,200 µmol photons m^−2^ s^−1^) to induce OCP-related PBS quenching (Supplemental Figure S1). Saturating flashes were administered to probe the maximum fluorescence level. In Figures 1, 6, we show the decrease of F_mb_′, maximum fluorescence under blue–light illumination. In vitro experiments were conducted in 0. 5 M phosphate buffer, pH 7.5. PBSs at a concentration of 0.012 μM were illuminated with strong blue–green light (900 µmol photons m^−2^ s^−1^) at 23°C in the presence of previously photoactivated OCP. The OCP-to-PBS ratio was set at 8 or 20.

#### Closure of PSII reaction centers

Reaction center closure was followed using a PSI ﬂuorometer (PSI Instruments, Brno, Czech Republic) in the 1-ms to 1-s time range, in dark-adapted (15 min) and quenched *Synechocystis* cells. The concentration of cells was set to 2.5 μg Chl/mL. WT and Δ*apcC* cells were illuminated with 1,200 μmol photons m^−2^ s^−1^ of blue–green light during 3 min to obtain quenched cells. Before measuring, DCMU (10 μM) and DBMIB (20 μM; Sigma) were added. Blue measuring light (*λ* = 460 nm) and orange actinic light (35 μmol photons m^−2^ s^−1^, *λ* = 630 nm) were used in all cases.

### GFP reporter assay and construction of the respective plasmids

To introduce the *apcZ* sRNA gene into the pZE12-luc plasmid, complementary primers Syr2_aqua_sense and Syr2_aqua_as (all primer sequences are shown in Supplemental Table S1) were annealed and introduced in pZE12-luc by AQUA cloning (Beyer et al., 2015). The backbone of pZE12-luc was amplified by inverted PCR using the primers PLlacoD and pZE_aqua_right, and the pJV300 control plasmid as template. The pZE12-luc plasmid containing syr2mod was generated by inverted PCR and subsequent AQUA cloning using the primers Syr2mod_fw and Syr2mod_rv, and the pZE12-luc_syr2 plasmid as template. The 5′-UTR of *slr1963* was optimized for protein expression by exchanging the native ribosome binding region AAAGTTGATTCACGTATAAAACCT with the ribosome binding region of pQE70 plasmid GAATTCATTAAAGAGGAGAAATTAAGC using annealing primers slr1963_RBS_aqua_sense and slr1963_RBS_aqua_as. The backbone of pXG10 (expressing sfGFP) was amplified by inverted PCR using the primers pXG10_sfGFP_aqua_righ and pXG10_sfGFP_aqua_left. Plasmids containing the 5′-UTR of slr1963_pQE70 and the 5′-UTR of slr1963_pQE70_RBS fused to sfGFP were generated by AQUA cloning (Beyer et al., 2015). The pXG10 plasmid containing the modified 5′-UTR of slr1963_pQE70_RBS was generated by inverted PCR and subsequent AQUA cloning using the primers slr1963mod_fw and slr1963mod_rev_new, and the pXG10_ slr1963_pQE70_RBS plasmid as template (Ochman et al., 1988).

In general, GFP assays were performed as previously described (Richter et al., 2010). Briefly, *E. coli* Top10 cells were transformed with the plasmids encoding the *slr1963* 5′-UTR fused to sfGFP and one of the sRNA encoding plasmids. The colonies were inoculated into 200 µL of antibiotic-containing LB medium and were grown overnight at 37°C in a 96-well plate with gentle agitation at 150 rpm in an air humidity saturated environment to prevent evaporation. Cells were diluted 1:10 into fresh LB medium and fixed in 1% HistoFix (Roth). Single-cell fluorescence was determined by flow cytometry using an Accuri C6 flow cytometer (BD Bioscience). Cell fluorescence was measured at an excitation wavelength of 488 nm and the emission was detected at 533 ± 15 nm. The mean fluorescence per plasmid combination was calculated from 50,000 events (cells) of 12 individual clones.

### Phylogenetic analysis

The phylogenetic analysis of ApcZ homologs in Figure 7, B was inferred using the maximum-likelihood method and Tamura–Nei model (Tamura and Nei, 1993) as implemented in MEGA7 (Kumar et al., 2016). Initial tree(s) for the heuristic search were obtained automatically by applying Neighbor–Join and BioNJ algorithms to a matrix of pairwise distances estimated using the Tamura–Nei model, and then selecting the topology with superior log likelihood value. The tree is drawn to scale, with branch lengths measured in the number of substitutions per site. The analysis involved 12 nt sequences as given in the alignment in Figure 7, A. The final dataset enclosed a total of 79 positions in the alignment.

## Accession numbers

Sequence data from this article can be found in the GenBank/EMBL data libraries under accession numbers *apcC*: AGF51564.1, *apcD*: AGF50575.1, *apcF*: AGF53208.1, *ocp*: AGF51876.1, *sgfp*: X96418.1. apcz is available in the Third Party Annotation Section of the DDBJ/ENA/GenBank databases under the accession number TPA: BK014370

## Supplemental data

**Supplemental Figure S1**. PAM fluorescence traces in WT and *ΔapcC* mutant cells.

**Supplemental Figure S2**. Coomassie Brilliant Blue-stained gel and immunoblot detection of OCP in Δ*apcC*, Δ*apcD*, Δ*apcF*, and *apcE-C190S* PBS mutants.

**Supplemental Figure S3**. 5′- and 3′-RACE experiments to compare the origins of ApcZ during exponential growth and during nitrate starvation and 3′-RACE experiments.

**Supplemental Figure S4**. Sequences of *apcC* and *apcZ* and position of oligonucleotides used for amplification of cDNA.

**Supplemental Figure S5**. Accumulation of ApcZ during nitrate starvation in WT and Δ*apcC* cells.

**Supplemental Figure S6**. OCP-induced decrease of fluorescence in PBSs isolated from Δ*apcZ* cells.

**Supplemental Figure S7**. LC–MS/MS analysis to demonstrate that APC is present in the PBS purified from Δ*apcZ* cells.

**Supplemental Figure S8**. Search for *acpZ* homologs in cyanobacteria lacking OCP.

**Supplemental Table S1**. Oligonucleotides used in this work

## Supplementary Material

koaa030_Supplementary_Data

## Notes

J.Z. and C.S. carried out the biochemical, photobiological, and molecular genetic experiments. J.Z. designed and constructed the mutants. I.S. performed the northern analyses. C.S. and W.R.H. performed the bioinformatics analyses. D.K. designed the study. D.K. and W.R.H. drafted the manuscript with contributions from all authors. All authors analyzed the data, and read and approved the final manuscript.

The author responsible for distribution of materials integral to the findings presented in this article in accordance with the policy described in the Instructions for Authors (https://academic.oup.com/plcell) is: Diana Kirilovsky (diana.kirilovsky@cea.fr).
