# Supplementary material for: Inverse regulation of light harvesting and photoprotection is mediated by a 3′-end-derived sRNA in cyanobacteria
Source: Plant Cell. 2020 Dec 14;33(2):358–80. doi: 10.1093/plcell/koaa030 (PMC8136909; doi:10.1093/plcell/koaa030)
Supplement: koaa030_Supplementary_Data [file koaa030_supplementary_data.zip › tpc.00491.2020-s02.pdf]

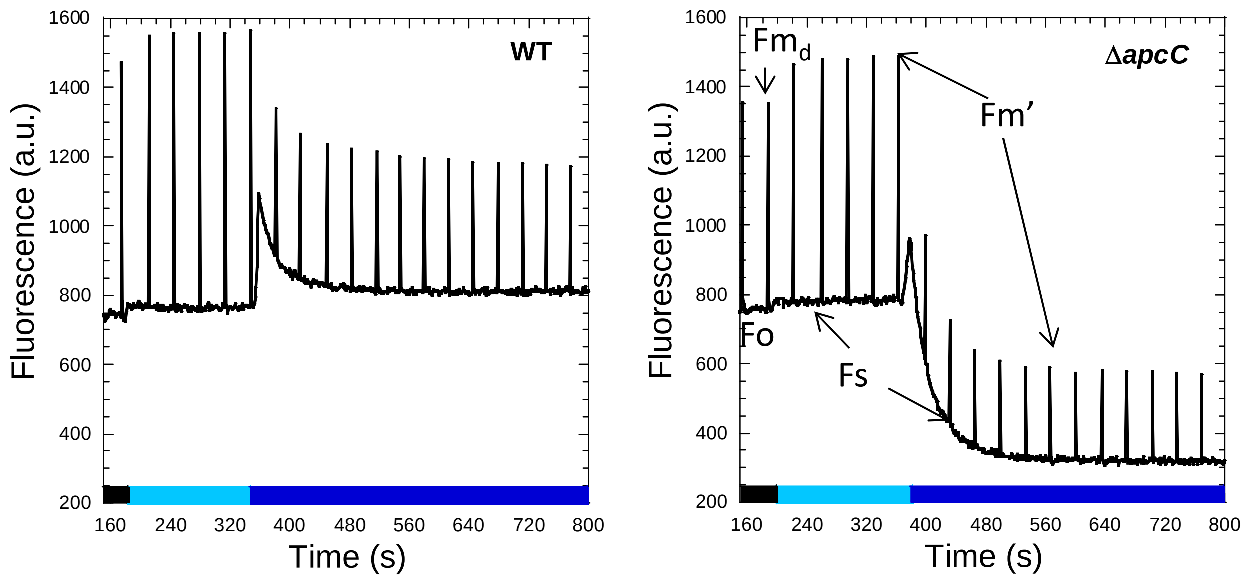

Supplemental Figure 1. PAM Fluorescence Traces in WT and Δ*apcC* Mutant Cells. (Supports Figures 1 and 6.)

The PAM fluorometer detects the fluorescence excited by a weak measuring beam consisting of a train of pulses at a frequency of 1.6 kHz. Changes in the fluorescence level reflect changes in fluorescence yield. In the PAM fluorometer, the minimal fluorescence level (Fo) is determined by using a low-intensity (nonactinic) red modulated light to illuminate dark-adapted cells. Under these conditions, all the centers are open: no measurable charge separation is induced. The maximal fluorescence level Fm_d (in the dark) and Fm' (under illumination) is measured by applying pulses of intense white light which transiently close all photosystem II centers thereby removing the photochemical quenching (qp). Under continuous illumination, the steady-state fluorescence level (Fs), which depends on the redox state of the quinone QA, is measured. The modulated-low-intensity-measuring light has a maximum of excitation at 650 nm and the fluorescence is detected at wavelengths beyond 700 nm. In cyanobacteria, this measuring light is absorbed by the phycobilisomes and the chlorophyll. As a consequence, a decrease in the fluorescence levels observed in a PAM fluorometer could be the result of a diminution of either the phycobilisome emission, or the chlorophyll-antenna emission, or caused by a decrease of the energy transfer from the phycobilisome to the PSII. The OCP-dependent decrease of fluorescence induced by strong blue light is mostly related to a quenching of phycobilisome fluorescence (Wilson et al, 2006).

Dark-adapted cells present a low Fm_d level characteristic of cyanobacteria, which are in state 2 due to the reduction of the PQ pool by respiratory substrates. Upon illumination by low intensities of blue light (80 µmol photons s^{-1} m^{-2}), exciting preferentially PSI, a maximal level of Fm' is reached. This increase of fluorescence is related to a state 1 transition induced by the oxidation of the PQ pool upon illumination. Subsequently, the exposure of cells to high intensities of blue light (1200 µmol photons s^{-1} m^{-2}) induced fluorescence quenching. All fluorescence levels (Fm', Fs and Fo) decrease. a.u.: arbitrary units

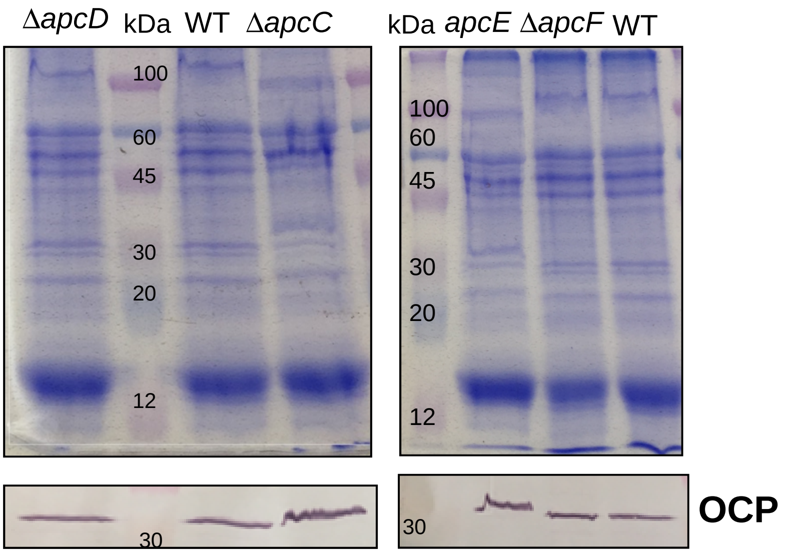

Supplemental Figure 2. Coomassie Brilliant Blue-Stained Gel and Immunoblot Detection of OCP in Δ*apcC*, Δ*apcD*, Δ*apcF* and *apcE-C190S* PBS Mutants. (Supports Figure 1D.)
The mutant constructions are described in Jallet et al. (2012) and Harris et al. (2016). Membrane-phycobilisome complexes containing all of the OCP present in the cells (Wilson et al., 2006) were purified from WT and mutants cells. The only PBS mutant that contains more OCP is Δ*apcC* and it is the only PBS mutant presenting more OCP-induced quenching**.**.

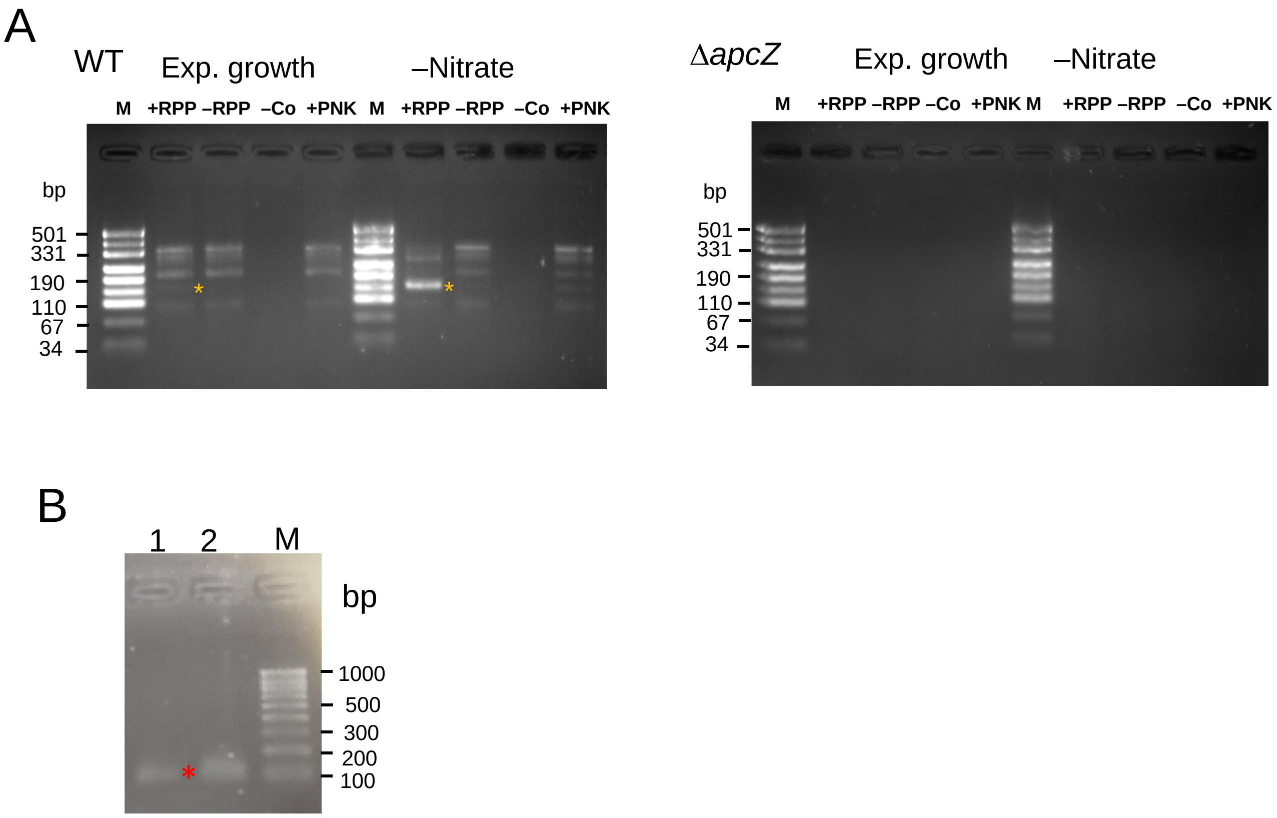

C

CAGCGCATCATGAAAATGGGCGGCAAAATTGTGAAAGTCGAATTAGCCACTGGCCGTCCC

GGCACCAATGCTGGTCTAGCCTAAGCTTGAGACTAGTTGTTGCTAATTTAGCA**ATGTTGA**

ATGGCAATTTTATGAATCAATTTTTTCGAGGTCAGCAATGGCCTCTTTTTTTGTTTGGT

***

Supplemental Figure 3. 5' and 3' RACE Experiments to Compare the Origins of ApcZ during Exponential Growth and during Nitrate Starvation. (Supports Figure 3.)
(A) 5' RACE experiments to compare the origins of ApcZ during exponential growth and during nitrate starvation. 5'RACE analysis using the same four parallel reactions as in Figure 3. The RNA either was treated with RppH (+RPP), enriching for 5' ends originating from a transcriptional start site but not selecting against monophosphorylated ends, or was not treated (-RPP), allowing ligation of the RNA linker only to preexisting monophosphorylated 5' ends. After treatment with PNK, also nonphosphorylated 5' hydroxyl ends were ligated. -Co, negative control without the 5' adapter and without any enzymatic treatment of RNA. Specific amplification products are labeled with asterisks. As input for this analysis, total RNA was prepared from WT cultures (left) or Δ*apcZ* for negative control (right). DNA of plasmid pUC19 digested by *Hpa*II served as size standard.
(**B**) 3' RACE experiments to determine the 3' end of *apcZ*. Amplification products of the 3 'RACE analyses using the specific primer 3' RACE-1 and 3′ linkerPCRrev primer in the first PCR, followed by a second PCR with nest primers 3' RACE-2 and 3′ linkerPCRrev primer. 1 and 2 are two independent replicates. **(C)** Sequencing of the chosen colonies. The PCR bands in parts (A) and (B) were excised and cloned. Ten colonies were screened by PCR and four of them were sequenced to determine the 3′ end. The sequence results are shown in red (3 of 4 clones). The ApcZ sequence based on 5' RACE and 3' RACE results is underlined in green and the *apcC* gene in black. The stars indicate the mapped 3' end.

A
```
TCCCGTTCTGTCGCATATGCCAACTTTTTAAGAGAAAACGTTTTAGGAGAGCCACACCCC
ATGCGGATGTTTAGAATTACGGCTTGTGTTCCTAGCCAAACCCGGATTCGGACACAACGG
GAATTACAAAATACCTATTTTACGAAGTTGGTGCCCTATGACAATTGGTTTCGTGAGCAA
CAGCGCATCATGAAAATGGGCGGCAAAATTGTGAAAGTCGAATTAGCCACTGGCCGTCCC
GGCACCAATGCTGGTCTAGCCTAAGCTTGAGACTAGTTGTTGCTAATTTAGCAATGTTGA
ATGGCAATTTTATGAATCAATTTTTTCGAGGTCAGCAATGGCCTCTTTTTTTTGTTTGG
```

B

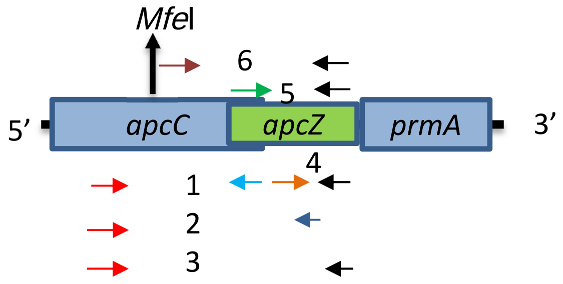

Supplemental Figure 4. Sequences of *apcC* and *apcZ* and Positions of Oligonucleotides Used for Amplification of cDNA. (Supports Figure 4.)

(A) The DNA sequence beginning 60 nt upstream of the *apcC* start codon to the last nucleotide of the *apcZ-prmA* intergenic spacer is given (position 1,431,638 to 1,431,995 on the forward strand of the *Synechocystis* chromosome sequence as given in GenBank accession BA000022.2). The blue and red boxes are the recognition sites of *Nde*I and *Mfe*I restriction enzymes, respectively. The *apcC* start and stop codons are boxed in purple, the *apcC* coding sequence is underlined in black and the sequence of *apcZ* (based on our 5’ and 3’ RACE results) is underlined in green.

(B) Scheme indicating the location of oligonucleotide primers used for RT-PCR. In parts (A) and (B), the positions of oligonucleotides are marked by arrows of the same respective color.

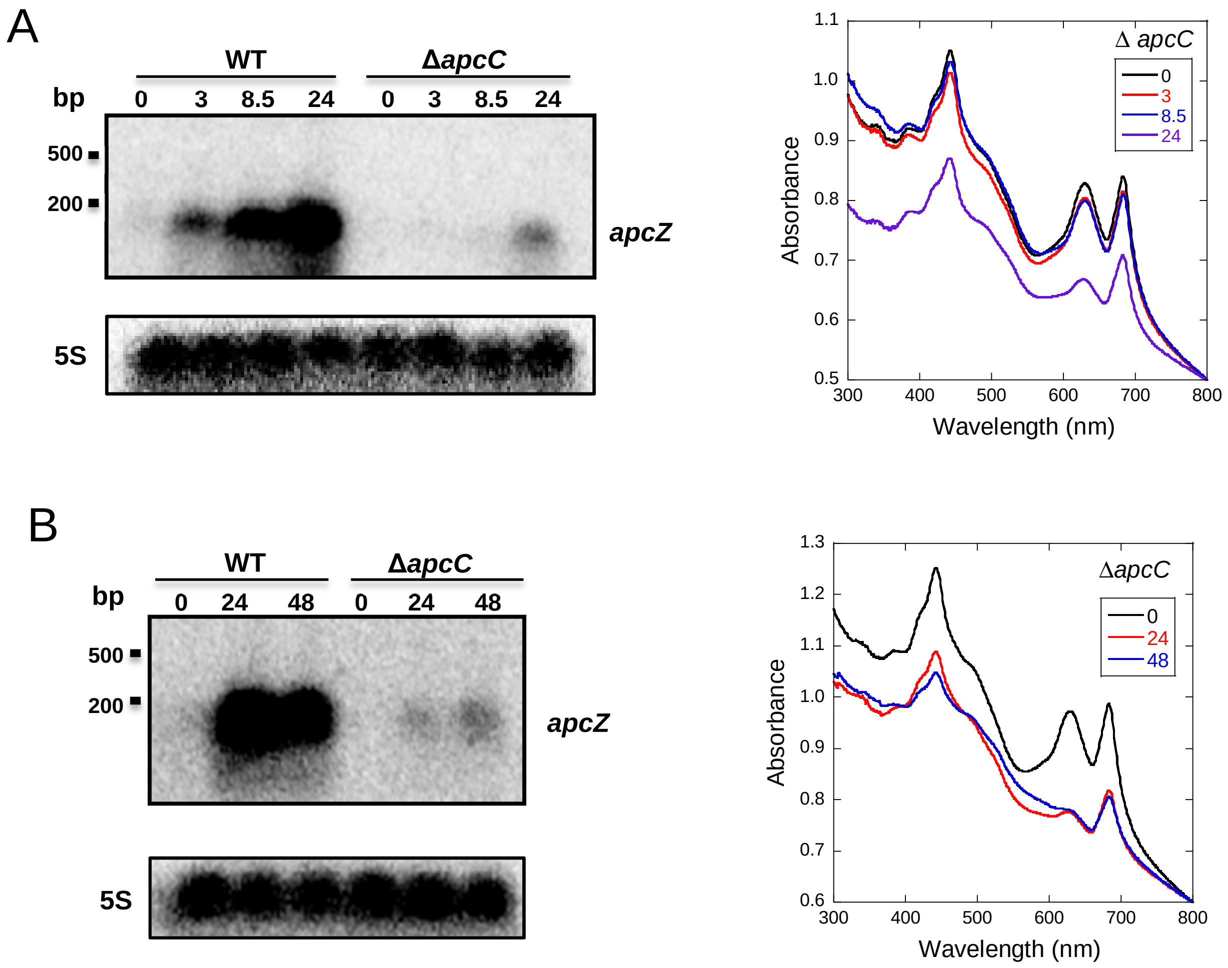

Supplemental Figure 5. Accumulation of ApcZ during Nitrate Starvation in WT and Δ*apcC* Cells. (Supports Figure 5.)

(A) and **(B)** RNA gel blot analysis of total RNA isolated from WT and Δ*apcC* cells grown in the absence of nitrate 0, 3, 8.5 and 24 h **(A)** or 0, 24 and 48 h **(B)**, using a ^{32}P-labeled transcript probe specific for ApcZ after electrophoretic separation on a 1.5% denaturing agarose gel. Hybridization for the 5S rRNA was used for a loading control. Absorbance spectra of Δ*apcC* cells during nitrate starvation are also shown. The 685-nm peak is related to chlorophyll *a* and the 635-nm peak is related to phycocyanin. In the experiment shown in (A) the decrease of Chl and PC in the Δ*apcC* mutant was slower than in the experiment shown in Figure 3. As a consequence also ApcZ was detected at later times and was visible at 24 h. In the experiment shown in (B) we observed that whereas in the WT the concentration of ApcZ is maximal already at 24 h, in Δ*apcC*, the amount of ApcZ continued to increase. Two biological independent experiments are shown in parts (A) and (B).

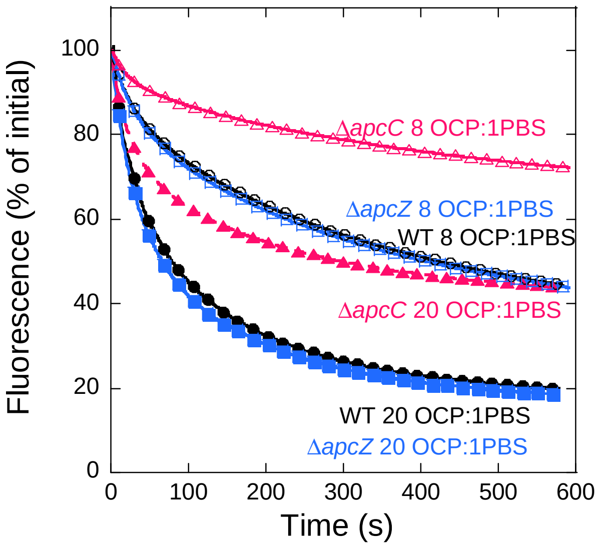

Supplemental Figure 6. OCP-Induced Decrease of Fluorescence in PBSs Isolated from Δ*apcZ* Cells. (Supports Figure 6.)

Purified PBSs (0.012 µM) from WT (black circles), mutant Δ*apcZ* (blue squares) and Δ*apcC* (fuchsia triangles) were incubated with prephotoactivated OCP (OCP to PBS ratio = 20 (closed symbols) and 8 (open symbols) in 0.5 M phosphate buffer at 23°C under strong blue light (900 µmol•m^{-2}•s^{-1}). The decrease of fluorescence was measured with a PAM fluorometer. The graph is the mean of three biologically independent experiments. For clarity, the error bars are not indicated in the figure. This figure indicates the presence of ApcC in the PBSs of the Δ*apcZ* mutant.

WT

Score	Mass	Matches	Pep(sig)	Sequences	Seq(sig)	emPAI	Description
3818	7857	130	130	8	8	86.78	ApcC_ ssr3383,
3328	9203	81	81	8	8	137.76	cpcD

Score	Mass	Matches	Match(sig)	Sequences	Seq(sig)	emPAI	Description
4884	7857	221	221	5	5	16.95	ApcC_ ssr3383
3839	9203	105	105	7	7	52.00	cpcD
444	23568	9	9	2	2	0.81	Bcaseine

Δ*apcC*

Score	Mass	Matches	Pep(sig)	Sequences	Seq(sig)	emPAI	Description
4152	9203	91	91	8	8	137.76	cpcD
159	7857	5	5	4	4	4.09	ApcC_ ssr3383,

Score	Mass	Matches	Match(sig)	Sequences	Seq(sig)	emPAI	Description
5758	9203	165	165	8	8	85.91	cpcD
428	23568	12	12	2	2	0.81	Bcaseine
192	7857	9	9	5	5	16.93	ApcC_ ssr3383 pl

Δ*apcZ*

Score	Mass	Matches	Pep(sig)	Sequences	Seq(sig)	emPAI	Description
5386	9203	119	119	10	10	196.38	cpcD
4261	7857	147	147	7	7	24.91	ApcC_ ssr3383,

Score	Mass	Matches	Match(sig)	Sequences	Seq(sig)	emPAI	Description
5781	7857	268	268	8	8	99.61	ApcC_ ssr3383
4925	9203	136	136	9	9	140.46	cpcD
238	23568	7	7	2	2	0.81	Bcaseine

Supplemental Figure 7. LC-MS/MS Analysis to Demonstrate that APC is Present in the PBS Purified from ΔapcZ Cells. (Supports Figure 6.)
Two biological independent preparations of the PBS of WT, Δ*apcC* and Δ*apcZ* were performed. The proteins of the PBSs were separated in an SDS gel and the protein strip from 5 kD to 15 kD was cut off for mass spectrometry analysis. The protein CpcD was used as a positive control because it is present in all the PBSs and β-caseine as a negative control. This figure indicates the presence of ApcC in the PBSs of Δ*apcZ* mutant and WT and its absence in Δ*apcC*.

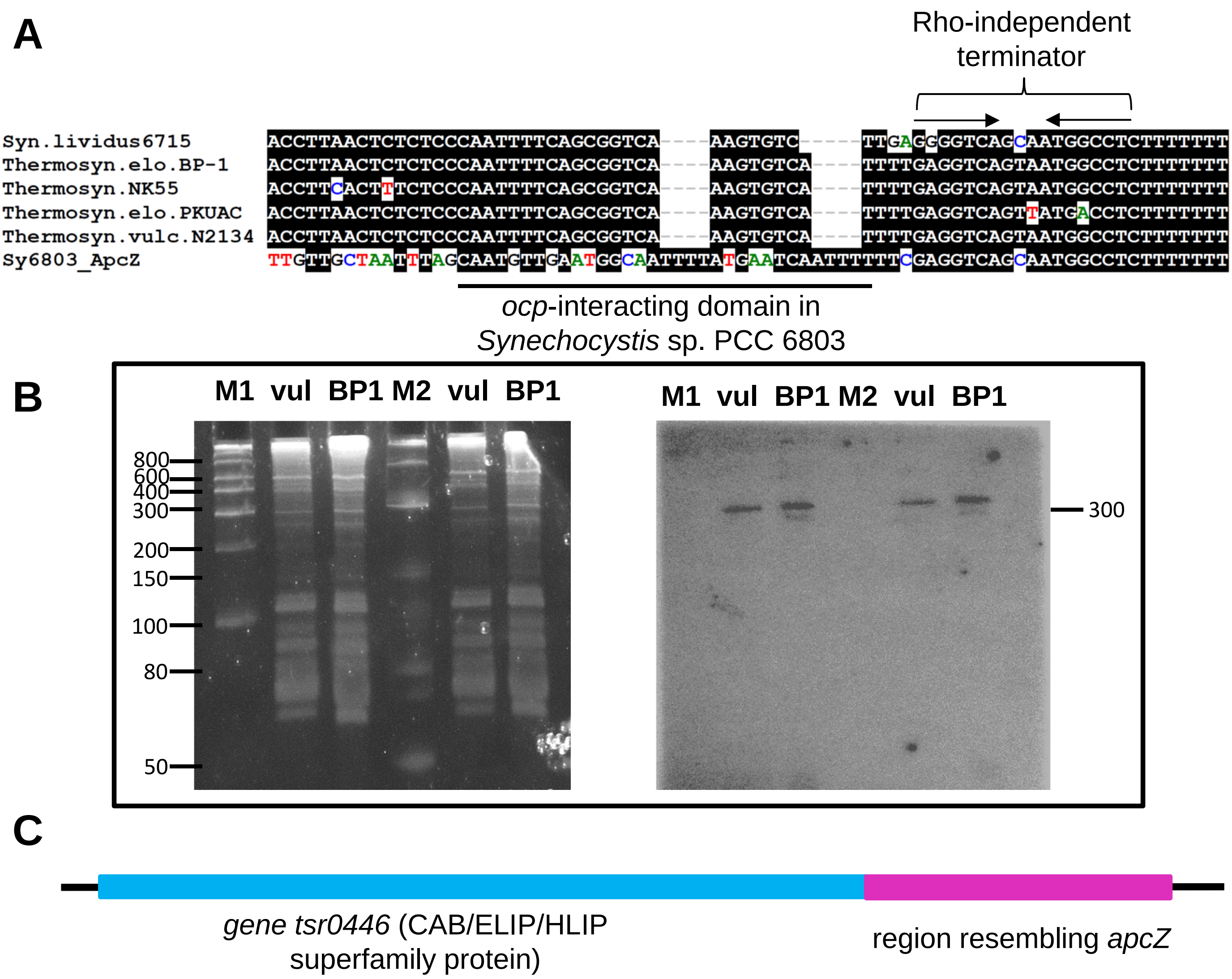

Supplemental Figure 8. Search for *apcZ* Homologs in Cyanobacteria Lacking OCP. (Supports Figure 7.)
(A) Alignment of possible candidates in five strains closely related to *Thermosynechococcus* with the *apcZ* sequence from *Synechocystis* at the bottom. **(B)** RNA gel image (left) and RNA blot hybridization (right) of a single-stranded RNA probe against the region from part (A) against replicate RNA samples from *Thermosynechococcus vulcanus* NIES-2134 (vul) and *Thermosynechococcus* sp. BP-1 (BP1). M1, M2, two different size markers. **(C)** Overview of the sequence arrangement in *Thermosynechococcus elongatus* BP-1. The region resembling *apcZ* is highlighted in fucsia.

Supplemental Table 1: Oligonucleotides Used in This Work.

Primer name	Sequence （5' to 3'）	Purpose
ΔSyR2-up-F	CGggatccCTCGGTGTACCCATCTCTTC[#]	ΔSyR2 construction
ΔSyR2-up-R	AGgatatcTTAGGCTAGACCAGCATTG[#]	
ΔSyR2-down-F	GGggtaccCCATATAGCGACCACCGTCC[#]	ΔSyR2 construction
ΔSyR2-down-R	GGggtaccCCCGTTGGCGATCGGTTT[#]	
SyR2-F	GGAATTCcatatgAGTCGAATTAGCCACTGG	SyR2 OE construction
SyR2-R	CGggatccTTCAGGCCAGAAGGGAAG	
3' RACE-1	GCTTGAGACTAGTTGTTGC	3'RACE/RT PCR
3' RACE-2	ATGTTGAATGGCAATTTTA	3'RACE
3' linker	AGATGAATGCAACACTTCTGTACGACTAGAGCA	3'RACE
3' linkerPCRrev*	GTGCTCTAGTCGTACAGAAGTGTTGCATTCATC	3'RACE
pXG10_sfGFP_aqua_right	GGATCCGCTGGCTCCGCTGCTGG	Amplification of pXG10 for aqua cloning
pXG10_sfGFP_aqua_left	TGTGCTCAGTATCTCTATCACTG	
PLlacoD	GTGCTCAGTATCTTGTTATCCG	Amplification of pZE12-luc for aqua cloning
pZE_aqua_right	CTAGAGGCATCAAATAAAACGAAAGG	
Syr2_aqua_sense	CAAGATACTGAGCACAGTTGTTGCTAATTTAGCAATGTTGAATGGCAATTTTATGAATCAATTTTTTCGAGGTCAGCAATGGCCTCTTTTTTTCTAGAGGCATCAAAT	Generation of syr2 insert for aqua cloning by primer annealing
Syr2_aqua_as	ATTTGATGCCTCTAGAAAAAAAGAGGCCATTGCTGACCTCGAAAAAATTGATTCATAAAATTGCCATTCAACATTGCTAAATTAGCAACAACTGTGCTCAGTATCTTG	
Syr2mod_fw	GTTGCTAATTTAGCGATCTTGAAAGGCAATTTT	Generation of syr2mod plasmid by inverted PCR
Syr2mod_rev	AAAATTGCCTTTCAAGATCGCTAAATTAGCAAC	
slr1963_RBS_aqua_sense	GATAGAGATACTGAGCACAGAATTCATTAAAGAGGAGAAATTAAGCatgccattcaccattgactctgcccgcGGATCCGCTGGCTCC	Generation of slr1963 UTR insert for aqua cloning by primer annealing
slr1963_RBS_aqua_as	GGAGCCAGCGGATCCGCGGGCAGAGTCAATGGTGAATGGCATGCTTAATTTCTCCTCTTTAATGAATTCTGTGCTCAGTATCTCTATC	
slr1963mod_fw	atgccTttcacGatCgactctgcccgc	Generation of slr1963mod plasmid by inverted PCR
slr1963mod_rev_new	GCGGGCAGAGTCGATCGTGAAAGGCATGCTTAATTTCTCC	
IG_1431930_for[$]	<u>TAATACGACTCACTATAGG</u>CAAACAAAAAAAGAGGCCATTGCTGACC	Generation of transcript probe for OcpR1
IG_1431930_rev	GACTAGTTGTTGCTAATTTAGCAATGTTG	
slr1963fw	GCCAACTCAATGCCGAAGATCAATTAG	Generation of transcript probe for slr1963
slr1963rev[$]	<u>TAATACGACTCACTATAGG</u>CAACTGGCGTAGGTGCGGC	

Supplemental Table 1, cont.

Primer name	Sequence （5' to 3'）	Purpose
apcC-F<br>apcC-R-1	GGATTCGGACACAACGGGAA<br>AGGCTAGACCAGCATTGGTGC	Primer pair 1 in Figure 4
apcC-F<br>apcC-R-2	GGATTCGGACACAACGGGAA<br>TAAAATTGCCATTCAACAT	Primer pair 2 in Figure 4
apcC-F<br>apcC-R-3	GGATTCGGACACAACGGGAA<br>GCCATTGCTGACCTCGAA	Primer pair 3 in Figure 4
3' RACE-1<br>apcC-R-3	GCTTGAGACTAGTTGTTGC<br>GCCATTGCTGACCTCGAA	Primer pair 4 in Figure 4
ocpR long-F<br>apcC-R-3	AGTCGAATTAGCCACTGGCCGT<br>GCCATTGCTGACCTCGAA	Primer pair 5 in Figure 4
apcC-F'<br>apcC-R-3	CGTGAGCAACAGCGCATCATGA<br>GCCATTGCTGACCTCGAA	Primer pair 6 in Figure 4
RNA oligo 1	AUAUGCGCGAAUUCCUGUAGAACGAACACUAGA<br>AGAAA	5' RACE RNA oligo
Syr2_RT	CATTGCTGACCTCGAAAAAAATTG	5' RACE RT primer
Adapt52	ATGCGCGAATTCCTGTAGAAC	5' RACE adapter primer
Adapt52_nest	AATTCCTGTAGAACGAACACTAG	5' RACE adapter primer for second PCR amplification
Syr2_NR	ATTCATAAAATTGCCATTCAACATTGC	5' RACE-specific primer
Syr2_NR2	CAACTAGTCTCAAGCTTAGGC	5' RACE nested specific primer
Syr2_NR_new	CTCGAAAAAAATTGATTCATAAAATTGCC	5' RACE nested specific primer 2
CP1	AGTCACGACGTTGTAAAACGACGG	Amplification of inserts in plasmid pGEMT by colony PCR
CP2	CAATTTCACACAGGAAACAGCTATGAC	

[#]Introduced restriction sites are in lower case.
*The linker was a 5' phosphorylated oligonucleotide blocked by a C6 aminogroup at the 3' end.
[$]The underlined sequence part corresponds to the T7 RNA polymerase promoter.

Supplemental File 1. Zhan and Steglich et al. (2021). Plant Cell. Inverse Regulation of Light Harvesting and Photoprotection Is Mediated by a 3' End-Derived sRNA in Cyanobacteria.

One-way analysis of variance; Bonferroni test

(OriginLab Corporation, OriginPro® 2020)

Overall ANOVA 1

	DF	Sum of Squares	Mean Square	F Value	Prob>F
Model	2	1.63E+08	8.17E+07	32.30566	1.69E-08
Error	33	8.35E+07	2530087.763		
Total	35	2.47E+08			

Fit Statistics

R-Square	Coeff Var	Root MSE	Data Mean
0.66192	0.19627	1590.62496	8104.3975

Bonferroni test

	MeanDiff	SEM	q Value	Prob	Alpha	Sig	LCL	UCL
slr1963_RBS-ApcZ slr1963_RBS-pJV300	-5206.32333	649.36992	-8.0175	8.98E-09	0.05	1	-6844.17255	-3568.4741
slr1963_RBS-ApcZmod slr1963_RBS-pJV300	-2279.60667	649.36992	-3.51049	0.00395	0.05	1	-3917.45588	-641.75745
slr1963_RBS-ApcZmod slr1963_RBS-ApcZ	2926.71667	649.36992	4.50701	2.35E-04	0.05	1	1288.86745	4564.56588

Overall ANOVA 2

	DF	Sum of Squares	Mean Square	F Value	Prob>F
Model	2	3.80E+08	1.90E+08	18.12551	4.88E-06
Error	33	3.46E+08	1.05E+07		
Total	35	7.26E+08			

Fit Statistics

R-Square	Coeff Var	Root MSE	Data Mean
0.52347	0.20979	3237.97051	15434.22361

Bonferroni test

	MeanDiff	SEM	q Value	Prob	Alpha	Sig	LCL	UCL
slr1963mod_RBS-ApcZ slr1963mod_RBS-pJV300	-1601.1325	1321.89592	-1.21124	0.70322	0.05	0	-4935.2355	1732.9705
slr1963mod_RBS-ApcZmod slr1963mod_RBS-pJ\	-7552.32917	1321.89592	-5.71326	6.74E-06	0.05	1	-10886.4322	-4218.2262
slr1963mod_RBS-ApcZmod slr1963mod_RBS-Ap	-5951.19667	1321.89592	-4.50202	2.38E-04	0.05	1	-9285.29966	-2617.0937
