# Supplementary figures and images for: Inverse regulation of light harvesting and photoprotection is mediated by a 3′-end-derived sRNA in cyanobacteria
Source: Plant Cell. 2020 Dec 14;33(2):358–80. doi: 10.1093/plcell/koaa030 (PMC8136909; doi:10.1093/plcell/koaa030)

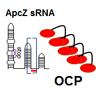

Supplement: koaa030_Supplementary_Data [file koaa030_supplementary_data.zip › tpc.00491.2020-s03.jpg]
