# Supplementary material for: Inverse regulation of light harvesting and photoprotection is mediated by a 3′-end-derived sRNA in cyanobacteria
Source: Plant Cell. 2020 Dec 14;33(2):358–80. doi: 10.1093/plcell/koaa030 (PMC8136909; doi:10.1093/plcell/koaa030)
Supplement: koaa030_Supplementary_Data [file koaa030_supplementary_data.zip › tpc.00491.2020-s01.docx]

**Dear Editor:**

We would be grateful if you would consider the revised version of our manuscript “Inverse regulation of light harvesting and photoprotection mediated by a 3’ end-derived sRNA in cyanobacteria” by Zhan et al. we are herewith submitting for publication in *The* *Plant Cell*.

We appreciate your decision to consider a revised version of our manuscript and thank the reviewers for their comments and suggestions. We also thank you for the possibility to have 90 days (instead of 60 days) to perform the suggested additional experiments.

Please see below our answers (in red) to your comments (in black). Separately, we answer to all the questions and comments of the three reviewers.

You wrote:

“We ask you to pay attention to the following points in preparing your revision:

1. The text would benefit from more extensive proofreading. You should clarify the questions being addressed, to place the work in context of the sRNA field, to correct inaccuracies and inconsistencies with the nomenclature, and more precise choice of wording. The Plant Cell reaches a broad audience of plant biologist. Hence, it would behoove you to pay particular care to tailor your presentation to the broadest cross-section of plant science researchers by embracing clarity and conciseness. Related to this, we might suggest that you add 'cyanobacteria' in the title, so that the readers aren't confused in scanning the titles that this is a land plant phenomenon.”

Reply: The manuscript was largely rewritten and we did an extensive proofreading. Since most of the text was changed and new figures were added we do not send a separate manuscript file with highlighted modifications. But we do hope that in the new text we have answered all reviewers’ questions satisfactorily.

We have rewritten the first section of the introduction to tailor it better towards a broader auditorium of plant scientists. We added another section into the discussion to place our results more deeply into the context of the sRNA field. Finally, we have added 'cyanobacteria' in the title.

“2. Reviewer 1 raises concern regarding the quantitation and better characterization of OcpR1 under different conditions, which should be addressed in a revised version. The editor concurs with the criticism that additional work is needed to make a more compelling argument for a promoter at the 5' end of OcpR1.”

Reply: The new version of our article has been thoroughly revised, considering all editorial and reviewer’s suggestions and performing additional experiments.

Following yours and reviewers’ remarks, we did quantification of RNAs and proteins and we performed new experiments:

1) Quantification of OcpR1 for **Figure 2**.

2) The coomassie blue stained gels of all the western blots in **Figures 1** and **6** and **Supplementary Figure S2** are shown as loading controls.

3) Quantification of OCP was added in **Figure 6**.

4) We further studied the accumulation and origin of OcpR1 under nitrate starvation conditions. New experiments were performed with WT and Δ*apcC* strains. We show absorbance spectra and northern blots resulting from these experiments in the new **Figure 5 and supplementary Figure 5** that allowed confirming that the free OcpR1 sRNA concentration increases during nitrate starvation not only in WT cells but also in Δ*apcC* cells.

5) In addition, the Δ*apcC* results support the presence of a promoter within the 3' region of *acpC.* We followed the appearance of free OcpR1 during nitrate starvation in the Δ*apcC* mutant. The northern blot clearly shows the increase of a band corresponding to free OcpR1 during the experiment (Figure 5B). In this mutant, OcpR1 cannot be a product of processing of the long *apcABC* mRNA since, due to the presence of an antibiotic cassette, the long mRNA includes only *apcA* and *apcB*. Thus, our results confirm the existence of newly synthesized OcpR1 in this mutant and the presence of OcpR1promoter in the 3’ half of the *apc* gene.

6) New 5’RACE experiments using RNA isolated from the Δ*apcC* strain shown in **Figure 5C** confirmed also this hypothesis: a low concentration of OcpR1 is present in Δ*apcC* cells; this concentration increases under nitrate starvation. The first nucleotide of this OcpR1 is the same than that of OcpR1 in WT.

We have adapted the respective sections in our article accordingly.

Please note that, following a comment by reviewer 2, we have replaced the designation of the here described sRNA OcpR1 by the new name ApcZ. It is much more common to name 3' derived UTR sRNAs after the mRNA they are made from (here the *apcABC* mRNA). Moreover, the same sRNA can have different targets in other organisms, so it could be misleading to call the sRNA according to the first detected target as described in this manuscript. The name was changed in the manuscript and in all figures and tables. However, in most of our answers to reviewer comments we kept the name OcpR1 for clarity.

Please, also see the point to point answer to all reviewers’ questions, comments and suggestions.

Also, please note the following:

-The Plant Cell now requires authors to complete and submit an author revisions checklist upon submission of a revised manuscript. The aim of the checklist is to aid authors in preparing a high quality manuscript, facilitate the review and assessment of revised manuscripts, and help to ensure that journal standards are maintained across the board. If your manuscript is accepted, the completed checklist will be published as supplemental material attached to the article online. Please download a copy of the checklist (pdf fillable form) at this link, for submission with your revised manuscript:

<https://tpc.msubmit.net/html/Author_Revisions_Checklist.pdf>.

Reply: the completed checklist was submitted with the revised version.

-Supplemental materials should be restricted to large datasets and tables, presentation of replicates, and validation of reagents, methods, or genotypes. Any data that are used to support the major claims must be in the main manuscript. Supplemental figure legends must indicate what figure in the main manuscript is supported by the supplemental data presented. Please justify how each of the supplemental figures meet the criteria.

Reply:

**Supplementary Figure S1**: PAM traces. Decrease of fluorescence induced by strong blue-green light. The figure relates to **Figures 1** and **6**.

**Supplementary Figure S2**: Immuno-blot detection of OCP in ∆*apcC*, ∆*apcD*, ∆*apcF* and *apcE-C190S* PBS mutants. The figure relates to **Figure 1**.

**Supplementary Figure S3**: 5’ RACE experiments to compare the origins of ApcZ during exponential growth and during nitrate starvation and 3’RACE experiments. The figure relates to **Figure 3**.

**Supplementary Figure S4**: Sequences of *apcC* and *apcZ* and position of oligonucleotides used for amplification of cDNA. The figure relates to **Figure 4**.

**Supplementary Figure S5**: Accumulation of ApcZ during nitrate starvation. The figure relates to **Figure 5**.

**Supplementary Figure S6**: OCP induced decrease of fluorescence in the PBS isolated from Δ*apcZ* cells. The figure relates to **Figure 6**.

**Supplementary Figure S7**: LC-MS/MS analysis to demonstrate that APC is present in the PBS purified from Δ*apcZ* cells. The figure relates to **Figure 6**.

**Supplementary Figure S8**: Search for *acpZ* homologs in cyanobacteria lacking OCP. The figure relates to **Figure 7**.

-Sampling methods and nature of "biological replicates" should be described precisely (i.e. different plants, parts of plants, pooled tissue, independent pools of tissue, sampled at different times, etc), along with a clear description of and rationale for any statistical analyses conducted. The reader should know exactly what was sampled; what forms the basis of the calculation of any means and statistical parameters reported. This is also necessary to ensure that proper statistical analysis was conducted.

Reply: In this work, we used cultures of unicellular cyanobacteria, thus pooled cells. When we talk about biological replicates it is meaning that the experiments were repeated using cultures grown on different days and the different experiments or RNA and protein extractions were done from these independent cultures.

When you are ready to submit the revised version, please upload a highlighted copy that indicates all changes made in response to the editor and reviewer recommendations. Include an itemized list of all changes made in response to each of the reviewer's suggestions in the "Response to Reviewers" section; please note that reviewers do not have access to your cover letter, nor was this decision letter shared with them.

Answer: Since we largely rewrote the manuscript (introduction, results and discussion), it is not productive to upload a copy with all the changes highlighted. Nevertheless, in the response to the reviewers we describe in detail all the changes that we did in the text and figures.

We are looking forward to hearing from you.

Yours faithfully,

Diana Kirilovsky and Wolfgang Hess
